# Application of RNAi to Genomic Drug Target Validation in Schistosomes

**DOI:** 10.1371/journal.pntd.0003801

**Published:** 2015-05-20

**Authors:** Alessandra Guidi, Nuha R. Mansour, Ross A. Paveley, Ian M. Carruthers, Jérémy Besnard, Andrew L. Hopkins, Ian H. Gilbert, Quentin D. Bickle

**Affiliations:** 1 Department of Infection and Immunity, London School of Hygiene and Tropical Medicine, London, United Kingdom; 2 Division of Biological Chemistry and Drug Discovery, College of Life Sciences, University of Dundee, Dundee, United Kingdom; University of Cambridge, UNITED KINGDOM

## Abstract

Concerns over the possibility of resistance developing to praziquantel (PZQ), has stimulated efforts to develop new drugs for schistosomiasis. In addition to the development of improved whole organism screens, the success of RNA interference (RNAi) in schistosomes offers great promise for the identification of potential drug targets to initiate drug discovery. In this study we set out to contribute to RNAi based validation of putative drug targets. Initially a list of 24 target candidates was compiled based on the identification of putative essential genes in schistosomes orthologous of *C*. *elegans* essential genes. Knockdown of Calmodulin (Smp_026560.2) (*Sm*-Calm), that topped this list, produced a phenotype characterised by waves of contraction in adult worms but no phenotype in schistosomula. Knockdown of the atypical Protein Kinase C (Smp_096310) (*Sm*-aPKC) resulted in loss of viability in both schistosomula and adults and led us to focus our attention on other kinase genes that were identified in the above list and through whole organism screening of known kinase inhibitor sets followed by chemogenomic evaluation. RNAi knockdown of these kinase genes failed to affect adult worm viability but, like *Sm*-aPKC, knockdown of Polo-like kinase 1, *Sm*-PLK1 (Smp_009600) and p38-MAPK, *Sm*-MAPK p38 (Smp_133020) resulted in an increased mortality of schistosomula after 2-3 weeks, an effect more marked in the presence of human red blood cells (hRBC). For *Sm*-PLK-1 the same effects were seen with the specific inhibitor, BI2536, which also affected viable egg production in adult worms. For *Sm*-PLK-1 and *Sm*-aPKC the *in vitro* effects were reflected in lower recoveries *in vivo*. We conclude that the use of RNAi combined with culture with hRBC is a reliable method for evaluating genes important for larval development. However, in view of the slow manifestation of the effects of *Sm*-aPKC knockdown in adults and the lack of effects of *Sm*-PLK-1 and *Sm*-MAPK p38 on adult viability, these kinases may not represent suitable drug targets.

## Author Summary

Schistosomiasis is a neglected tropical disease affecting 200 million people in the poorest areas of the world. To date no vaccine exists against this parasitosis, and the only possible cure relies on the use of the one available drug, praziquantel. Praziquantel is a safe and effective drug active against adult worms in a single dose administration. However the increasing threat of emerging resistance makes the search for novel drugs a compelling priority. *S*. *mansoni* possesses a quite large and complex genome so new *in silico* strategies to select genes which might represent potential novel drug target candidates are an important approach to drug development. Here, we report application of RNA interference, which knocks down gene function, to validate a number of putative drug targets for schistosomiasis. Some of these genes were selected because they had been shown to be essential for life in another worm species, *C*. *elegans*, and others because they were potential targets of compounds shown to be active against schistosomes *in vitro*. Although none of the identified genes looked promising as drug targets, our results showed important roles for some of the genes in schistosome development.

## Introduction

Schistosomiasis is a parasitic disease caused by a trematode of the genus *Schistosoma*, affecting around 200 million people in the poorest areas of the world and despite progress in development of a vaccine against schistosomiasis, none is yet available [1–3]. Therefore, currently, both treatment and most disease control initiatives [4] rely on chemotherapy using a single drug, praziquantel (PZQ), which is active against adult worms of all the medically important *Schistosoma* species [5]. Moreover, it has proved to be generally safe and effective using a single oral dose [6, 7]. However, there have been a number of reports of poor *in vivo* efficacy of PZQ [8–11] and strains isolated from such cases have shown lower susceptibility to PZQ experimentally [12] although as yet there is no convincing evidence of development and selection of heritable resistance even following repeated rounds of treatment [9, 13–15]. However, its increasingly extensive use, especially in mass drug administration programs, raises concerns about drug resistance emerging and this has led to renewed interest in research into drug discovery including: development and application of whole organism screens for compound testing [16]; rational drug discovery [17]; and identification of putative molecular targets by analysis of the annotated schistosome sequences [18].

Following the availability of transcriptome data and the complete genome sequence of *Schistosoma mansoni* [19–23] a number of groups have set out to identify likely drug targets from amongst the >11,000 genes predicted for the *S*. *mansoni* genome and to prioritise them for validation by molecular/biochemical techniques that can offer a quicker and more selective tool to identify a subset of possible essential genes. As recently reviewed [24] this *in silico* analysis has highlighted various “druggable” targets [25] in schistosomes [19, 26–28] and the application of comparative genomics has identified orthologues in schistosomes of druggable genes shown to be essential in other organisms. Such approaches have led to the compilation of a number of partially overlapping lists of putative targets [19, 20, 29]. The demonstration that effective gene knockdown can be achieved in a variety of life cycle stages of schistosomes using RNA interference (RNAi) [24, 30–36] has led to this method being applied to validate putative drug targets based on changes to phenotype and/or viability in culture or *in vivo* [32, 36–44].

In parallel with our ongoing whole organism high throughput screen (HTS) approach [45] we have also undertaken studies aimed at target identification and validation using RNAi. At the outset we were interested to compare the use of adult and larval schistosomes with a view to possible use of our automated image-based HTS [45] for drug-induced damage to larval schistosomes to develop RNAi HTS. Studies were initiate by selecting putative essential genes in *S*. *mansoni* which were orthologues of genes previously shown, through RNAi methodology, to be essential in *C*. *elegans* [46]. Moreiver the RNAi-induced phenotype of *Sm-*Protein Kinase C together with the highlighted potential of kinases as key targets for drug design in schistosomes [28, 47, 48] led us to focus on kinases in our bioinformatics list. We also identified some other potential targets known to interact with known small molecule, drug-like compounds (i.e. “druggable” targets). Early detection was based on compounds found to be active in whole organism screening of a focused kinase inhibitor library (from GlaxoSmithKline) and a diverse collection of lead-like compounds from the Division of Biological Chemistry and Drug Discovery, University of Dundee.

Thus, a number of additional potential kinases were selected and targeted for silencing in adult and larval stages to establish their essentiality. Overall, phenotypic effects were seen following the dsRNA-mediated knockdown of 4 out of 16 genes (Smp_026560.2, Smp_096310, Smp_009600 and Smp_133020 in SchistoDB) coding for: Calmodulin, atypical Protein kinase C, Polo-like kinase 1 and p38-MAPK family member, respectively.

## Materials and Methods

### Essentiality predictions by orthology

Orthologues were detected using OrthoMCL [49] version 1.4. The program was installed locally and modified to use the NCBI BLAST+ program [50]. The default OrthoMCL parameters, for e-value and MCL inflation index were used, as it had been shown previously [51] that tightening these parameters only increased specificity at a cost on sensitivity. OrthoMCL produced clusters of genes within the two given genomes representing co-orthologous groups. Where the cluster contained a single gene from each genome, these genes were considered true orthologs.

Where a cluster contained one gene from a species and multiple genes from the other species, the single gene was considered the ortholog, and the multiple genes considered paralogs. Where a cluster contained multiple genes from both genomes, all the genes were considered paralogs to each other.

### Identification of putative targets kinases from phenotypic screening of kinase inhibitor libraries

#### Datasets

We also used an additional chemocentric approach to identify potential drug targets in the schistosome kinome. We tested the GlaxoSmithKline Published Kinase Inhibitor Set (PKIS) against adult schistosomes [16]. The PKIS comprises 367 published small molecule kinase inhibitors that inhibit a range of kinases and represent a number of different chemical scaffolds. A number of compounds found to be active against larval and adult schistosome worms during screening of a collection of compounds from the Drug Discovery Unit (DDU), University of Dundee [45] were also used to explore potential drug targets. However, the DDU set does not contain experimental information on target activity.

### Predicted target assignment for the DDU set

In order to obtain some ideas about the potential targets that the DDU set may be targeted a multi categorical Laplacian-modified naïve Bayesian model, as described by Nidhi et al. [52] with ChEMBL version 6 as training set [53], using Pipeline Pilot v8.0 (Biovia) was built. Compounds from ChEMBL were considered active if they displayed an activity (Ki, EC50, Kd, Kb) below 10 μM and targets were kept if they had at least 10 active compounds, the dataset contains 254,253 compounds and 1,028 targets. The descriptors used in the model were molecular weight, ALogP, numbers of hydrogen bond donors, acceptors and rotatable bonds, fractional Topological Polar Surface Area (TPSA/total surface area) [54] and Extended-connectivity fingerprints of depth 6 (ECFP_6) [55]. For every compound the top prediction from the model was calculated and assigned as potential target of interest for that particular compound.

### Analyzing and visualizing experimental results

To visualize the results for the 2 sets of compounds, similarity networks were drawn where nodes are compounds and edges linked two similar compounds (one network per set). The similarity cut-off used is a Tanimoto score [56] higher than 0.7 using ECFP_4 as descriptors [55]. The networks were displayed in Cytoscape [57]. Individual sub networks were then analyzed to identify those which were enriched in in-vivo active molecules and to identify known or predicted targets of interest (S1 and S2 Figs).

### Ethics statement & animals

Experimentation was carried out using the NC3Rs and ARRIVE guidelines. It was approved following local ethical review by the LSHTM Animal Welfare and Ethical Review Board and was performed in strict accordance with the U. K Home Office Animals (Scientific Procedures) Act 1986 (approved H.O. Project License 600456).

Female CD1 mice (aged 6–8 weeks) supplied by Charles River, UK were maintained at St Mary’s Hospital, Imperial College London in SPF conditions with access to food and water *ad libitum*.

### Parasite preparation & maintenance

Experiments were performed using the Puerto Rican strain of *S*. *mansoni* maintained in *Biomphalaria glabrata* and CD1 mice. Schistosomula were mechanically prepared as previously described [45] using medium 169 (M169) [58] with 5% FCS. For some experiments the M169 was supplemented with 0.25% packed A+ human red blood cells (hRBC) (National blood transfusion service, Collindale, UK).

Mice were infected subcutaneously under mild isoflurane (Merial Animal Health Ltd (UK) anaesthesia with 400 cercariae in 100μl water. Adult worms were recovered from infected mice using sterile techniques by portal perfusion 6–8 weeks post-infection using warm perfusion medium (Dulbecco’s Modified Eagle’s Medium [DMEM], 2mM L-glutamine, 100u/ml penicillin, 100μg/ml streptomycin, 20mM Hepes, 10units/ml heparin [Sigma, UK] [16].

We used DMEM since this was the medium used in our previous studies [16] and has also been used by others for long term culture [59, 60]. As in these earlier studies we found good viability using cDMEM but would also expect M169 which we used for the schistosomula to work equally well [24, 61].

In some experiments the mice were infected with male only worms. These were produced by infection of snails with single miracidia and the cercariae produced screened by PCR using the female-specific W probe [62] and only male cercariae were used for mice infections. Adult parasites were washed free of red blood cells using the perfusion medium and finally placed in culture in complete medium (cDMEM: DMEM, 2mM L-glutamine, 100u/ml penicillin, 100μg/ml streptomycin, 10% foetal calf serum (FCS) at 37°C, in an atmosphere of 5% CO_2_ [59].

### dsRNA synthesis

For all the targets above, specific T7 promoter-tagged primers were designed in order to amplify a PCR product of ~500 base pair (bp) for each gene (S1 Table). We also produced an exogenous (irrelevant [IRR]) non-schistosome dsRNA fragment from the yeast expression vector pPIC9K as previously described [42]. The sequences of the primers were tested against the *S*. *mansoni* genome using the BLAST program at National Center for Biotechnology Information (NCBI) to avoid the risk of possible off-targeting. PCR products were used to synthesize *in vitro* the long dsRNAs using the T7 Megascript RNAi Kit (Ambion) following the instructions reported by the manufacturer. Briefly, all synthesis reactions were carried out for 4 hours at 37°C incubation followed by DNAse and RNAse treatments. At the end dsRNA products were run in a 1% agarose gel to check their integrity and concentrations assessed at OD_260_ using the ND 1000-Spectrophotometer.

In addition, for each target short interfering RNAs (siRNAs) were synthesized commercially from both Applied Biosystem (AB) and Integrated DNA Technology (IDT) which use a different algorithm to design them. From both companies two siRNAs were designed based on different regions of the sequence in order to avoid any possible annealing problems due to mRNA secondary structure. A siRNA irrelevant control was obtained from IDT based on pPIC9K sequence, as mentioned above.

### dsRNA delivery in schistosomes

#### Adult worms

Fifteen adult worms (males or pairs) were electroporated in 100 μl of electroporation buffer (Applied Biosystem) in 4 mm cuvettes and at specific conditions (125 V, 20 ms and room temperature) as previously described [34]. Worms were treated either with long dsRNA (100μg/ml) or using the synthetic siRNA (2.5μg/ml). After electroporation parasites were cultured overnight in cDMEM with 5% FCS and the following day the medium was replaced with cDMEM with 10% FCS which was subsequently changed once a week.

#### Schistosomula

In preliminary experiments simple incubation was compared with electroporation for delivery of dsRNA [63] but the incubation method was chosen for the experiments described herein. For this 10,000 schistosomula were incubated in 50 μl of M169 or RPMI medium without FCS for 30 minutes at 37°C in presence of long dsRNA (100 μg/ml). After the treatment parasites were transferred to 1 ml of complete M169 alone or supplemented with hRBC provided by addition of 2.5μl/ml packed, washed human whole blood cells. Medium±hRBC was changed once a week. The level of mRNA suppression was measured 7 days after the silencing by qRT-PCR. Adult worms were kept in culture for observation up to 8 weeks and schistosomula up to 6 weeks. During this time parasites were checked weekly for any phenotypic change (motility, viability and tegumental damage). All of the silencing/phenotype monitoring experiments were repeated at least twice and several three times.

### Gene expression analysis

To assess the level of gene knock down RNA was extracted from parasites using the Trizol method (Invitrogen) following the instructions reported by the manufacturer. Parasites were homogenized on ice using a sterile electronic pestle and the RNA precipitated overnight in ethanol, sodium acetate (3M, pH 5.2) and glycogen (1 μg/ml) and subsequently treated with DNase (Invitrogen) to remove any possible genomic DNA residues. cDNA was synthesized using 500 ng of total extracted RNA from each sample in the Super Script III kit (Invitrogen) and an oligo (dT)_20_ primer. Quantitative real-time PCR was performed in triplicate using custom TaqMan Gene Expression Assays including a specific set of primers and a probe labeled with 6-carboxyfluorescin (FAM), obtained from Applied Biosystem. All TaqMan probes have been designed using a gene sequence outside the region in which the long dsRNA was synthesized. PCR reactions were carried out in triplicate on a 7500 ABI PRISM Sequence Detection System Instrument using an equivalent of 10 ng of parasite RNA according to the manufacturer’s instructions. For relative quantification, the ΔΔ*C*_*t*_ method was employed, using alpha tubulin as the endogenous standard for each sample [64]. Results obtained from parasites treated with irrelevant dsRNA were used as calibrators [65]. For graphical representation, the ΔΔ*C*_*t*_ values were normalized to controls and expressed as percent difference.

### Infection of mice with dsRNA-treated schistosomula

Newly transformed schistosomula were soaked in presence of specific long dsRNA or control as described above. After the treatment parasites were maintained in culture overnight in 1 ml of complete M169 without blood. The following morning parasites were washed three times with M169 without serum, counted and resuspended in an appropriate volume to contain 1000 schistosomula/100 μl of medium. This was injected into CD1 mice (5 age and weight matched mice per group) maintained under mild isoflurane (Merial Animal Health Ltd (UK) anaesthesia. In order to increase the chance of administration of comparable numbers of larvae to each mouse, two separate intramuscular injections each of 50 μl suspension were administered into different thigh sites of each mouse using a 30 g needle (BD Micro-Fine^TM^+). Samples of the treated parasites were kept in culture in order to assess gene knock down 7 days and 28 days after the dsRNA treatment. Mature worms were recovered from mice 4 weeks later by mesenteric vein perfusion [66] and checked for number and morphological changes. Parasites were then used to assess the gene suppression levels by qRT-PCR as described above.

### Inhibitor treatment of parasites

500 newly transformed schistosomula were cultured in wells of 48 well plates (Nunc,UK) in 1 ml of M169 medium supplemented with 100 U/ml Penicillin, 100 μg/ml Streptomycin and 5% foetal calf serum (Sigma, UK) in the presence of 100 nM of BI2536 inhibitor (Axon MedChem, Netherlands) dissolved in DMSO (final DMSO concentration was 0.1%). Control schistosomula were cultured in medium with 0.1% DMSO. For adult worms, cultures were set up in 24 well plates (Nunc, UK) and 5 worm pairs recovered from mice 6 weeks post infection added to each well in 2 ml cDMEM. As above, 100nM of Bl12536 was added to test wells and DMSO to controls. Cultures were set up with or without 2.5μl/ml packed washed human hRBC. Parasites were maintained in 5% CO_2_ at 37°C and the medium ± inhibitor ± hRBC renewed twice a week.

### Statistical analysis

Differences between groups were assesses for statistical significance using unpaired Student’s t-test using GraphPad Prism 4.0 Software. Results are considered significant if *p* value < 0.05.

## Results

### Selection and target validation for putative drug targets

Genes predicted to be both essential and druggable (*i*.*e*. interact with small molecule ligands) in *S*. *mansoni*, were selected for study using RNAi. Due to the lack to whole genome essentiality studies in *S*. *mansoni* essentiality was inferred from *C*. *elegans* data, as the genome sequence [67] and a systematic functional analysis of the *C*. *elegans* genome, using RNAi, are available [46]. The latter study assigned each gene to a functional class based on the phenotype, and the functional classes used to define “essential” genes here were: (i) Embryonic lethality (Emb), defined as >10% dead embryos; (ii) Sterile (Ste), required a brood size of <10 (wild-type worms under similar conditions typically have >100 progeny); (iii) Sterile progeny (Stp), progeny brood size of <10. Gene essentiality in *S*. *mansoni* was inferred from the *C*. *elegans* data [46] using a logical model: the predicted gene has an ortholog or out-paralogs, but no in-paralogs and any one of the predictor co-orthologs has been experimentally verified as essential. The assumptions of the model were that if a gene was retained after a speciation event, despite a large evolutionary time and adaption to new conditions, then its function was potentially essential. However, if the gene had subsequently been duplicated there was a potential redundancy of function and the gene was not individually essential.

Of the 11,809 genes in the *S*. *mansoni* genome, 323 were predicted to be essential using this method. The 323 putative essential *S*. *mansoni* genes were searched against the protein targets in ChEMBL [53] (http://www.ebi.ac.uk/chembl/) using BLAST+, (E-value cut-off of 1×10^-03^ and target coverage of >50%). Only ChEMBL targets that had at least one potent compound (<10nM) were considered. This filter reduced the *S*. *mansoni* set to just 24 genes (S2 Table). Interestingly these genes cover a variety of functional categories including kinases, phosphatase and proteasome subunits. One of those selected genes, glycogen synthase kinase 3, GSK-3, (Smp_008260.1) was previously proposed as a potential drug target in *S*. *mansoni* [68].

### Selection of putative targets kinases from phenotypic screening of kinase inhibitor libraries

The 2 libraries of compounds were tested and the DDU set similarity network with active and inactive compound is shown in S1 Fig, while the activity of the PKIS dataset in-vivo are shown in S3 Table and the network similarity network in S2 Fig. Some sub networks were identified as being enriched in active compounds and the associated known or predicted targets for those compounds were investigated.

Some of the corresponding genes in the *S*. *mansoni* genome were selected by searching for the homologous gene directly in the schisto DB database (http://schistodb.net/schisto/) (i.e. EGFR). Other genes have been identified via information relative to the genes function and/or mechanism of action available in literature (i.e. Insulin receptor and PLK1). For all the other genes, the protein sequences of the human genes were used to query the schistosome genome via a BLAST search and the genes having the highest sequence similarity were selected. In this way a total of 10 different genes were selected for RNAi (some examples are indicated in S2 Fig). Despite effective mRNA knockdown no differences between dsRNA-treated and controls were observed for any of the genes tested in adult worms (S4 Table). However the silencing of 2 of the genes (Smp_133020 and Smp_009600) led to phenotypic changes and loss of viability when performed in larvae as described below.

In the first part of this work we present the results obtained from the silencing of several genes selected from S2 Table. In order to establish and confirm the optimal conditions to perform the RNAi in adult and larval parasites we started with the gene at the top of the predicted essential list, a putative calmodulin gene (Smp_026560.2).

Then, in the second part of this study we focused on kinases selected from phenotypic screening of kinase inhibitor libraries.

### Suppression of Calmodulin (Smp_026560.2) (*Sm*-Calm) affects adult worm morphology

**Fig 1:**
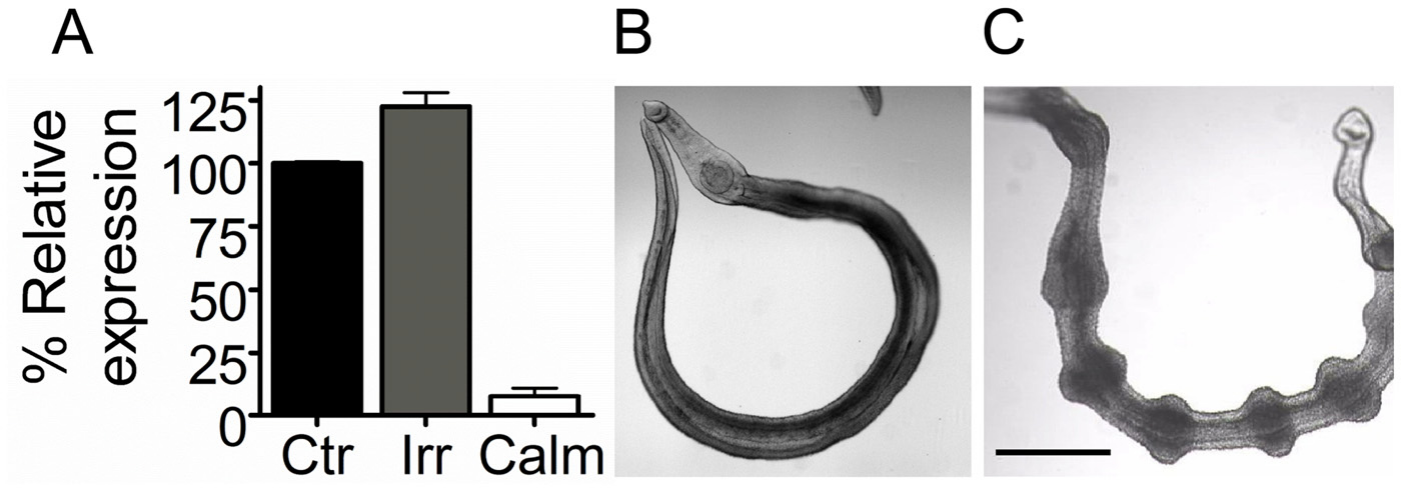
Effect of *Sm-*Calm suppression on adult schistosomes. (A) *Sm-*Calm expression in adult schistosomes 7 days after electroporation with *Sm-*Calm long dsRNA (CALM) relative to that in untreated control worms (CTR) or worms similarly electorporated with irrelevant dsRNA (IRR). (B-C) Light microscope images of adult worms electroporated 3 weeks earlier with IRR dsRNA (B) or *Sm-*Calm long dsRNA (C). Note the waves of contraction along the bodies of worms following *Sm*-Calm knockdown (scale bar = 100μm). Data are representative of 3 independent experiments.

Initial experiments involved electroporation of adult male worms in the presence of dsRNA since we and others had previously found this to be successful [34, 69]. We tested the ability of specific long *Sm-*Calm dsRNA (dsRNA) versus synthetic small interfering RNA (siRNA) (designed and supplied by Applied Biosystems [AB] and Integrated DNA Technology [IDT]) to efficiently suppress *Sm-*Calm expression. A week after the RNAi treatment relative gene expression was determined using qRT-PCR. We found that all dsRNA constructs exerted strong transcript level suppression, ranging from 70 to 95% compared to controls. Comparable knockdown was achieved with both ds and siRNA from two different companies used separately or in combination (S3 Fig). This and subsequent comparisons of ds and siRNAs with seven other genes showed both to be effective but in general the dsRNA gave somewhat higher levels of inhibition and, therefore, in later experiments we only used long dsRNA (normally ~500bp). Fig 1A shows data representative of 3 repeat experiments.

Relative expression of *Sm-*Calm a week after the electroporation with dsRNA was reduced by 90%. Between 1–2 weeks after treatment the worms started to manifest the characteristic waves of contraction/dilation (Fig 1C). In contrast control worms treated with irrelevant dsRNA (IRR) had a smooth and regular shape (Fig 1B). This phenotype did not seem to affect worm viability initially but by 5 weeks, when the waving phenotype had reduced, the treated worms appeared darkened, had damage to the tegument and showed lower motility compared with controls. Two months after the silencing *Sm*-Calm was still 50% lower compared to controls. Electroporation of a mixture of male and female worms, some in copula, resulted in mRNA suppression of 89% in male worms but only 26% in females. The contraction phenotype was not seen in the females but did occur in the males even when *in copula* although this did not seem to affect pairing. The same phenotype was seen with the siRNA suppression of *Sm-*Calm suppression.

### Optimisation of the conditions to perform RNAi in schistosomula

Whilst evaluating the effects of *Sm*-Calm RNAi in schistosomula we carried out numerous experiments to confirm the optimal protocols based on various methods reported in the literature: use of dsRNA delivery (by soaking or electroporation), age of parasites (newly transformed or one week old cultured schistosomula), the effect of different culture media on efficiency of knockdown and parasite survival) and culture with and without human red blood cells (hRBC). These findings, replicated with several other genes, are reported in brief since similar conclusions have been reached by others [24, 34, 39, 40] i.e. markedly better survival in M169 than in RPMI, better survival following delivery of dsRNA by soaking than electroporation, comparable mRNA knockdown in newly transformed and 7 days old schistosomula whether by electroporation or soaking. Based on these results the soaking method with newly transformed schistosomula using M169 was used for all further experiments. Although marked *Sm-*Calm suppression (up to >95%) was consistently observed, no phenotypic changes were observed in terms of larval survival or development when silenced parasites were cultured in M169 with or without hRBC.

### Suppression of atypical Protein Kinase C (Smp_096310) (*Sm*-aPKC) affects viability in both adult and larval schistosomes

#### Adult worm silencing

**Fig 2:**
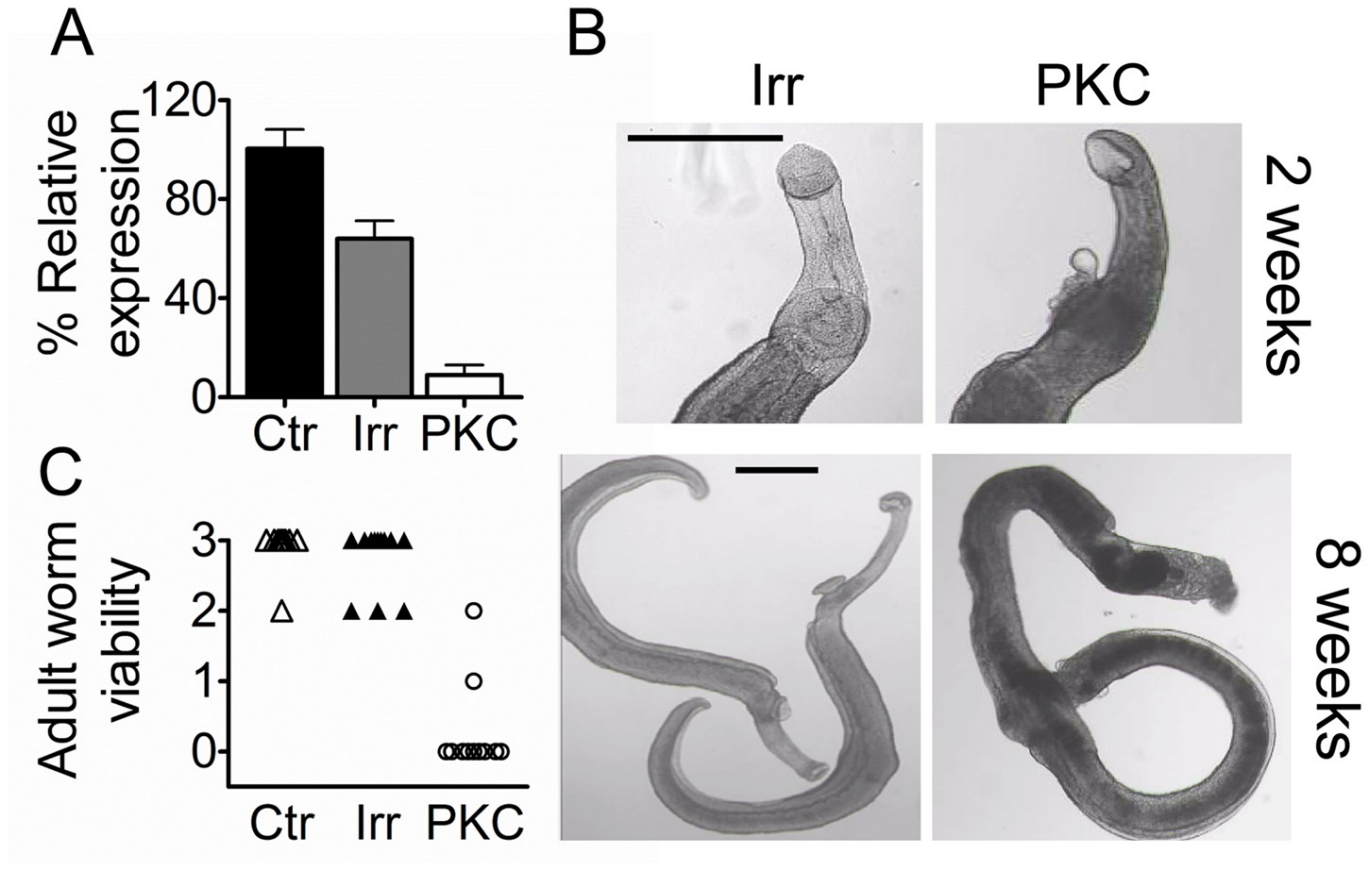
Effect of *Sm*-aPKC suppression on adult schistosomes. (A) *Sm-*aPKC expression in adult schistosomes 7 days after electroporation with *Sm-*aPKC long dsRNA (PKC) relative to that in untreated control worms (CTR) or worms similarly electroporated with irrelevant ds RNA (IRR). (B) Light microscope images of adult worms 2 or 8 weeks following electroporation with IRR or *Sm*-aPKC (PKC) long dsRNA (scale bars = 100μm). (C) Visual score of worm viability 3 weeks after silencing:- 3-healthy, attached; 2-healthy, not attached; 1- darkened/low motility; 0-severely damaged. Data are representative of 3 independent experiments.

The second gene in S1 Table was an atypical protein kinase C (*Sm-*aPKC). Fig 2A shows robust suppression (95%) of *Sm-*aPKC mRNA levels assessed by qRT-PCR a week after electroporation with dsRNA.

Suppression (range of 67–75%) was also achieved with siRNA from AB and IDT. *Sm-*aPKC silenced and control worms treated with IRR dsRNA or siRNA were visually similar during the first two weeks of culture but by day 14 the *Sm*-aPCK treated worms were noticeably darker than the controls, did not attach to the plate with their suckers and showed blebbing notably at the anterior end (Fig 2B). By the third week the presence of vesicles was more generalised and some worms showed severe damage. At this stage parasites were visually scored, assigning to each worm in culture a score from 0 to 3 (Fig 2C). By the 8^th^ week from the silencing the majority of the *Sm*-aPKC dsRNA-treated parasites appeared dead or severely damaged (Fig 2C). The same phenotype and kinetics were observed with the siRNAs. The data shown is representative of three independent repeats.

### Schistosomula silencing

**Fig 3:**
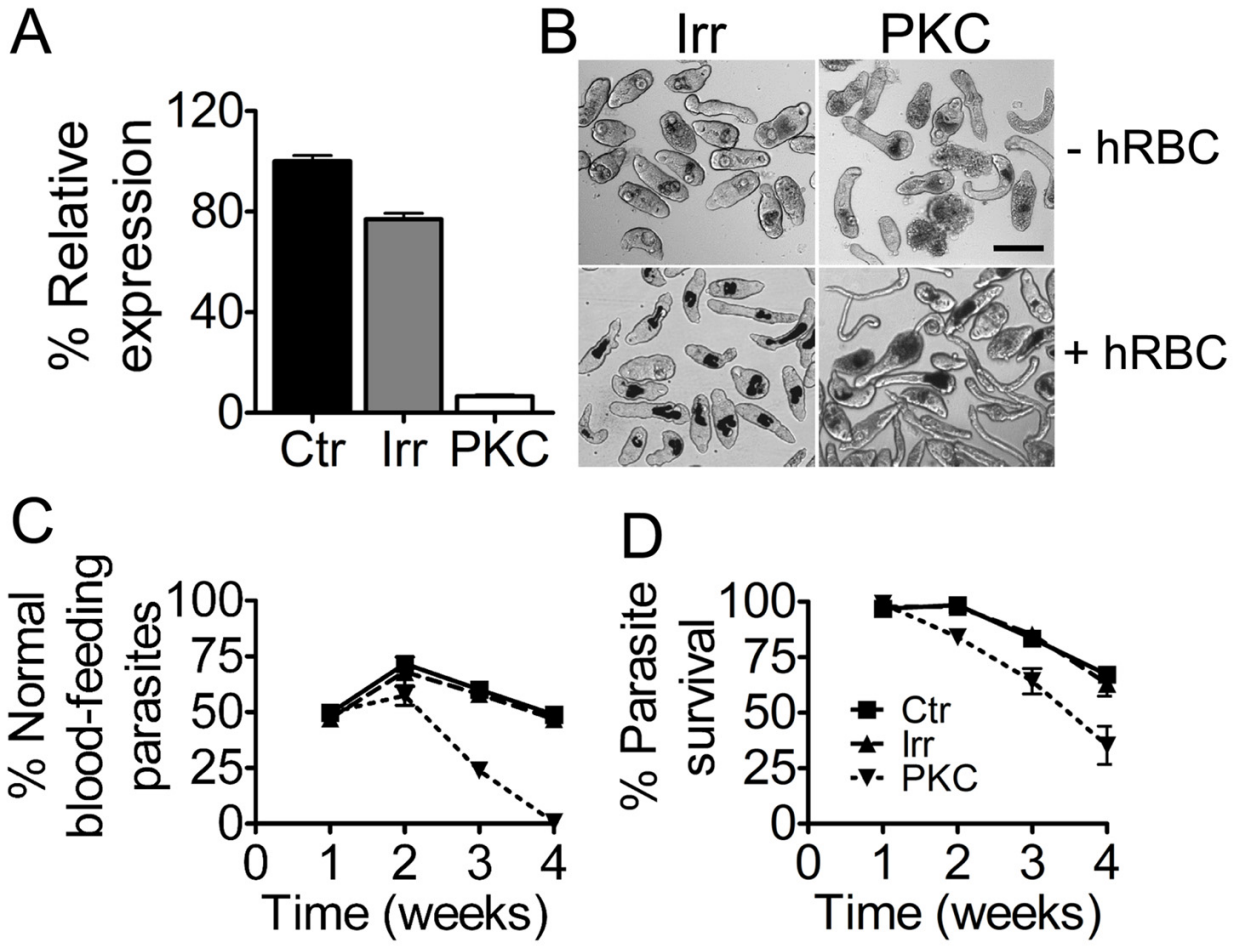
Effect of *Sm-*aPKC suppression in schistosomula. (A) *Sm-*aPKC expression in schistosomula 7 days after soaking with *Sm*-aPKC long dsRNA, irrelevant ds RNA (IRR) or untreated controls (CTR). (B) Light microscope images of schistosomula 4 weeks after the RNAi treatment. Top images:—wells without hRBC; bottom images:—wells with hRBC (scale bar = 100μm). Percent normal development (C) and percent survival (D) in cultures with hRBC (mean±SE triplicate cultures). Data are representative of 3 independent experiments.

High levels of mRNA knockdown were also achieved with schistosomula as seen in Fig 3A.

Initial experiments showed modest effects on schistosomula viability and so further experiments tested cultures with or without hRBC, since this triggers larval development and transcriptional changes in gene expression [70]. In the presence of hRBC the phenotypic effects were much more marked (Fig 3B). The control and *Sm*-aPKC treated schistosomula looked similar up to one week of culture with many ingesting hRBC and showing haematin pigment in their developing guts. After 2 weeks there was increased death of the *Sm*-aPKC treated schistosomula (Fig 3D) and a marked drop in the proportion of those with developing guts so that virtually all remaining by 4 weeks showed the elongate shape of the lung stage schistosomula (Fig 3C). This indicated high mortality once development had been initiated by onset of blood feeding *in vitro*. The data shown is representative of three independent experiments.

Due to the effects induced by silencing *Sm-*aPKC and the fact that kinases are considered to be an important class of potential drug targets, owing to the essential roles many of them play [28, 47] we focused our RNAi experiments on some other kinases in the list of putative essential genes i.e. caseine kinase (Smp_180400), serine/threonine protein kinase (Smp_080730), putative serine/threonine-protein kinase vrk (Smp_141380) and glycogen synthase kinase 3 (Smp_008260.1). As can be seen from S5 Table high levels of mRNA reductions were observed in adults for each of the genes although levels for the larvae were more variable. However, knockdown of none of these genes caused any phenotypic changes in either adult or larval stages.

### RNAi suppression of Polo-like kinase 1, *Sm*-PLK1 (Smp_009600), has no effect on adult viability but affects viability and development of schistosomula

*Sm*-PLK1 suppression experiments were performed three times using adult male and female worms cultured with or without RBC. Despite high levels of silencing (>90% mRNA reduction) no phenotypic changes were observed during culture for 5 weeks.

**Fig 4:**
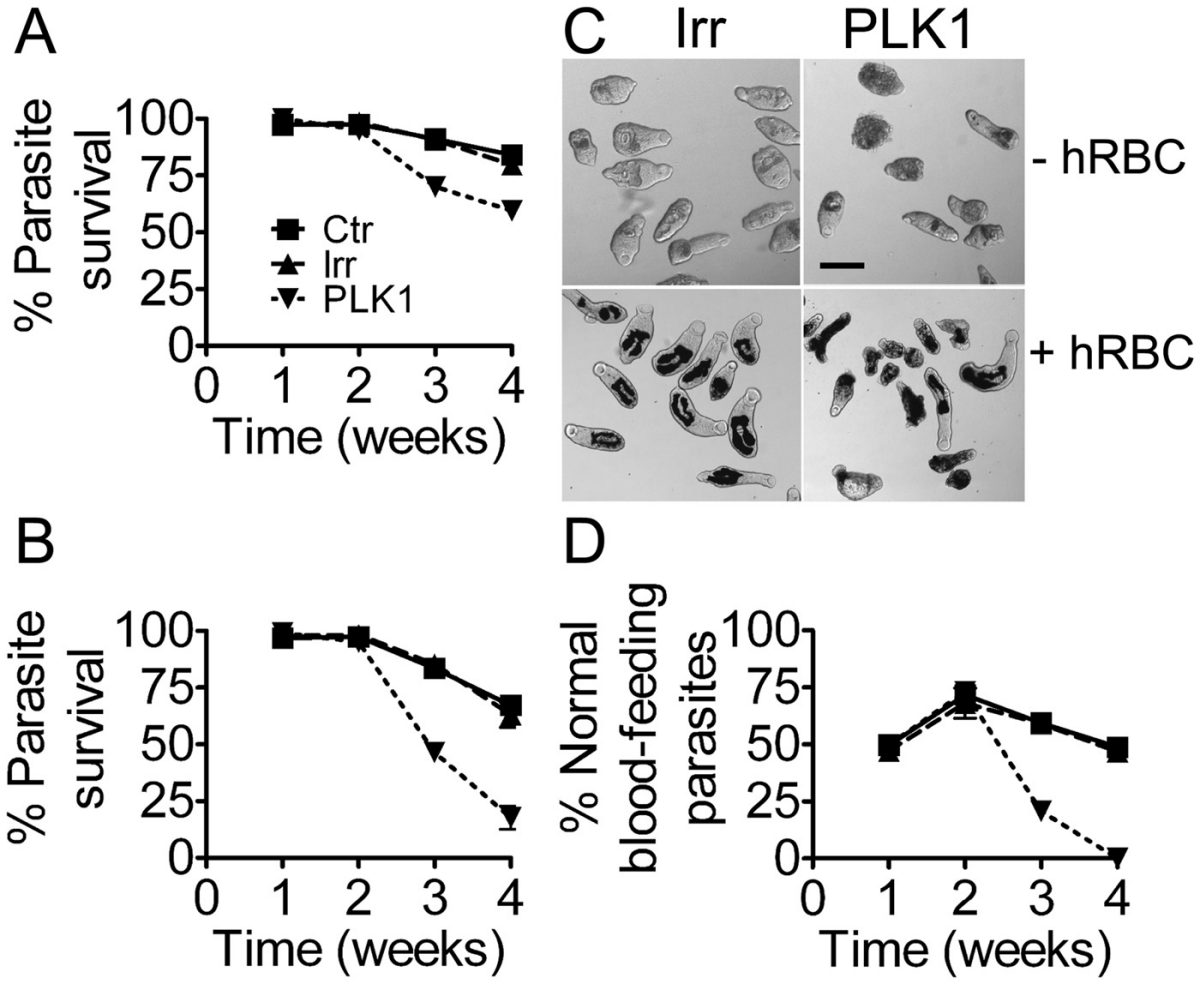
Effect of *Sm-*PLK1 silencing on schistosomula viability. (A-B) Survival of parasites treated with *Sm*-PLK1 long dsRNA compared with untreated controls (CTR) or controls treated with irrelevant dsRNA (IRR) in the absence (A) or presence (B) of hRBC. (C-D) Reduced viability and a lower percentage of blood feeding parasites in the presence of hRBC following *Sm*-PLK1 knockdown. The photomicrographs (C) are of schistosomula cultured for 3 weeks after RNAi treatment (scale bar = 100μm).

RNAi treatment of schistosomula induced >95% suppression of mRNA for *Sm*-PLK1 as assessed one week later. As shown in Fig 4 this silencing led to severe morphological abnormalities and reduced survival between two and three weeks of culture.

As with *Sm*-PKCa, this effect was markedly more pronounced with schistosomula cultured with hRBC, with only 15% of those in hRBC surviving at 4 weeks (Fig 4B) compared with 60% cultured without hRBC (Fig 4A).

### Inhibition of *Sm*-PLK1 with the specific inhibitor BI2356 affects schistosomula development/viability and also egg development

**Fig 5:**
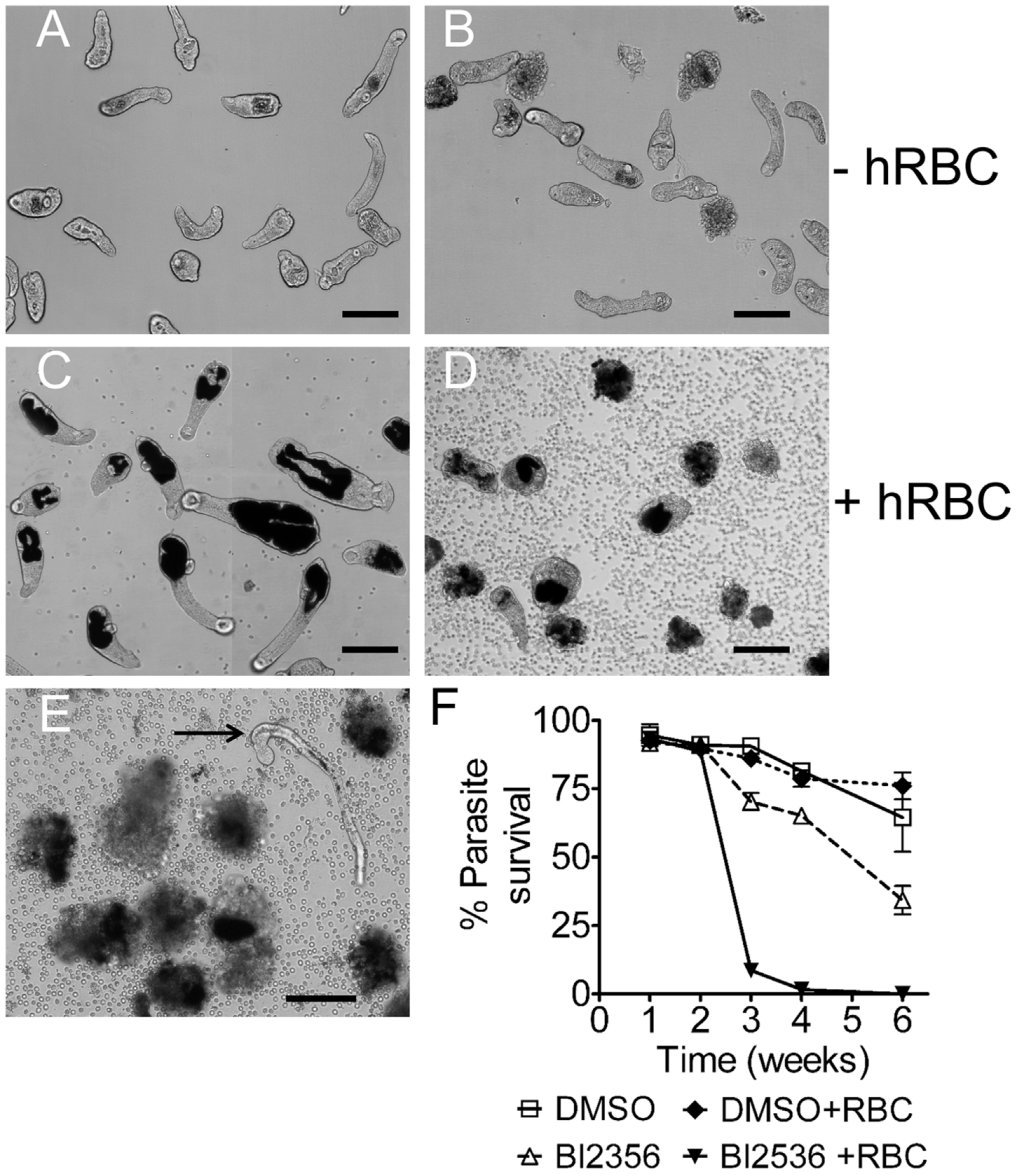
Effect of the PLK-1 inhibitor BI2536 on larval development. Schistosomula were treated with 100 nM BI2356 (B, D and E) or carrier DMSO alone (A and C). A and B: cultures without hRBC; C, D and E: cultures with hRBC. Images A-D: after culture for 20 days; image E: after culture for 30 days (arrow shows a viable lung-form schistosomulum) (scale bar = 100μm). (F) Mean ± SE larval survival based on triplicate cultures.

The above evidence of involvement of *Sm*-PLK1 in early worm development was corroborated by using the specific inhibitor, BI2536, as previously used with adult worms [71]. Triplicate cultures of 500 schistosomula with or without hRBC were set up as above in 48-well plates with or without 100nM Bl2536. During the first 2 weeks the inhibitor caused no obvious effect but by 3 weeks a proportion of larvae in the cultures with inhibitor showed morphological damage and death. This was markedly more pronounced in the cultures with hRBC (Fig 5) such that by 4 weeks very few remained alive and those that did were larvae which retained the lung form and had not started to feed (e.g. Fig 5E).

**Fig 6:**
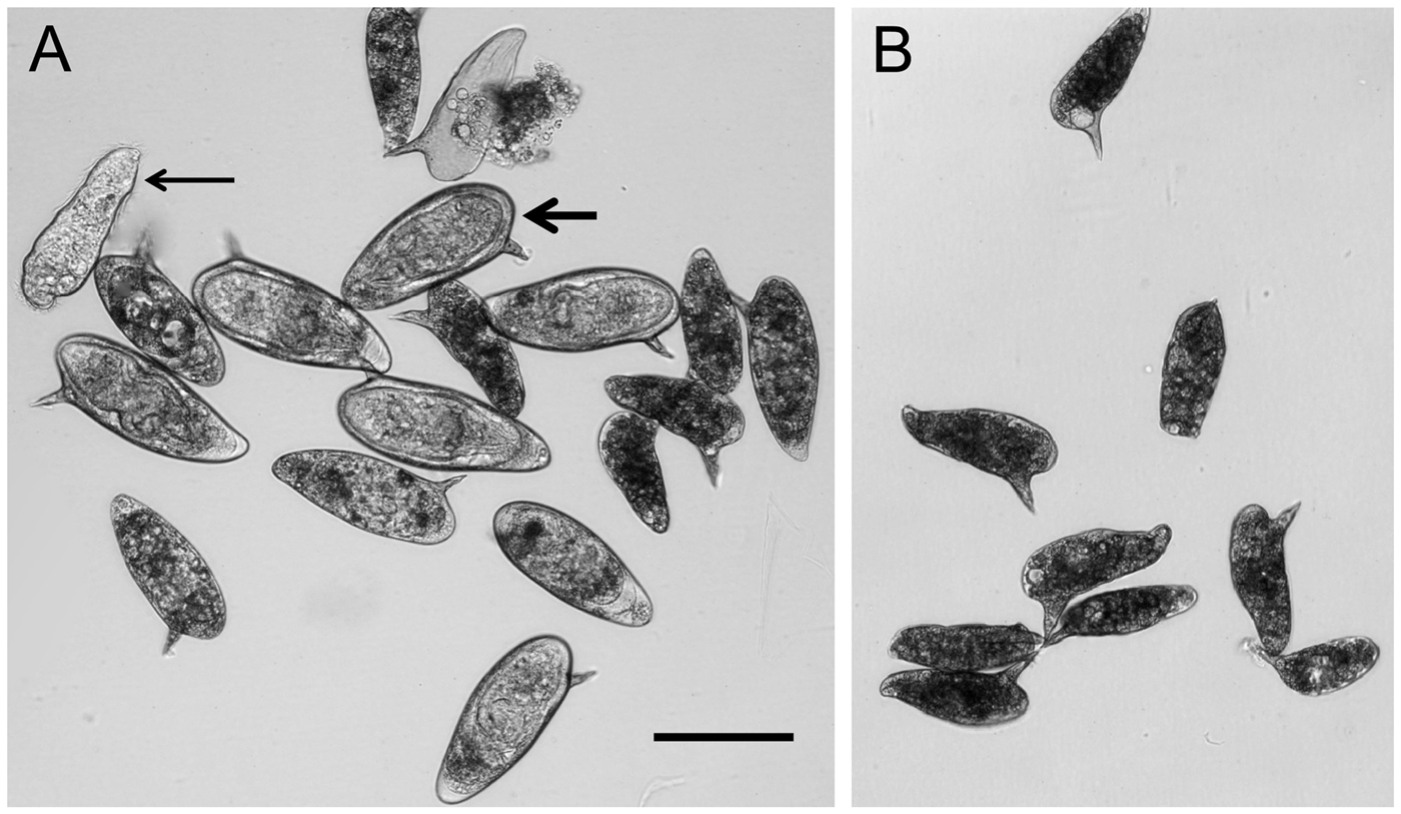
Effect of the PLK-1 inhibitor BI2536 on egg development. Unembryonated eggs recovered from *ex-vivo* adult worms and cultured in vitro for 3 days failed to embryonate when subsequently cultured in 100 nM BI2536 (B) in contrast most eggs cultured with DMSO carrier alone developed normally (A). Thin arrow: free miracidium; thick arrow: embryonated egg containing viable miracidium. Scale bar = 100μm.

BI2536 at 100nM was also used in adult worm cultures. During culture for 7 weeks no differences were seen in the morphology, behaviour or viability of the worms with or without inhibitor. However, in contrast to the controls the eggs produced in cultures with the inhibitor failed to embryonate. To investigate if this could be due to effects of the inhibitor on egg development, the unembryonated eggs produced by *ex-vivo* adults maintained for 3 days in control medium were recovered from the cultures and incubated with or without inhibitor in quadriplicate. After 10 days 41±9.3% of eggs in control cultures contained viable miracidia or had hatched whereas none of the eggs in the inhibitor had developed to contain miracidia (Fig 6). This clearly indicates that PLK-1 is necessary for embryonation of viable eggs.

### Schistosomula viability and development is inhibited by suppression of the p38-MAPK family member (Smp_133020)

**Fig 7:**
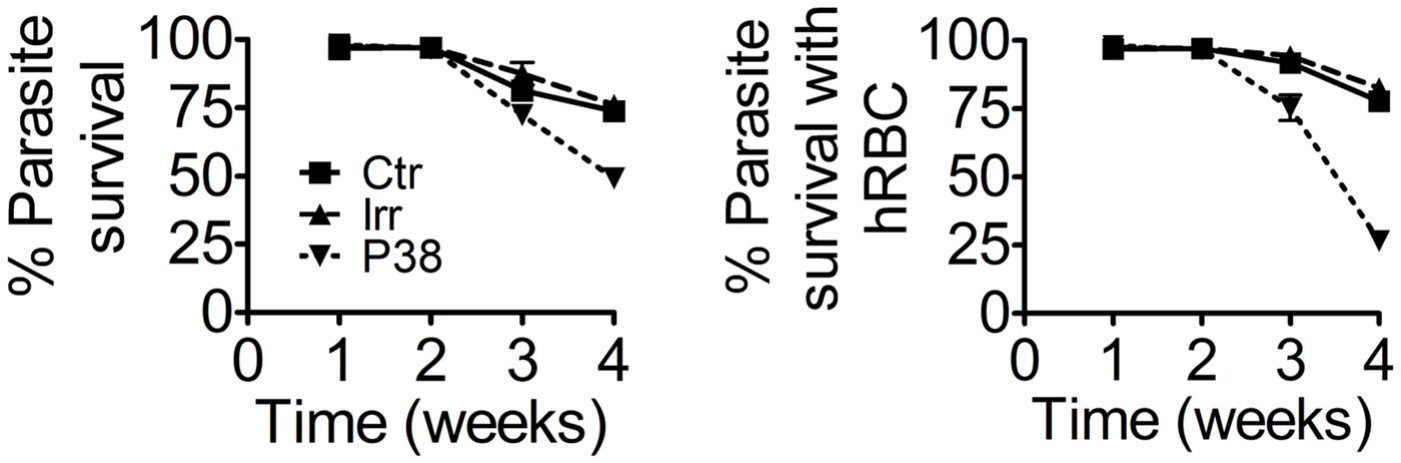
Effect of RNAi silencing of *Sm*-MAPK p38. Graphs showing percentage survival of schistosomula following treatment with long dsRNA corresponding to the *Sm*-MAPK p38 family member (Smp_133020) (P38) compared with untreated controls (CTR) or controls treated with irrelevant dsRNA (IRR). Schistosomula were cultured in the absence (A) or presence (B) of hRBC.

Marked suppression (>85%) of p38-MAPK mRNA was observed a week after the long dsRNA treatment in both adult worms and schistosomula. Again no phenotype was seen in the adult worms but the RNAi silencing consistently reduced viability of schistosomula starting after 2 weeks and, as previously observed for *Sm-*aPKC and *Sm-*PLK1, such effects on parasite viability were more evident when p38-MAPK silenced schistosomula where cultured in the presence of hRBC (Fig 7).

### Suppression of atypical Protein Kinase C (Smp_096310) (*Sm*-aPKC) and Polo-like kinase 1 (Smp_009600) (*Sm*-PLK1) affects viability of schistosomula *in vivo*

**Fig 8:**
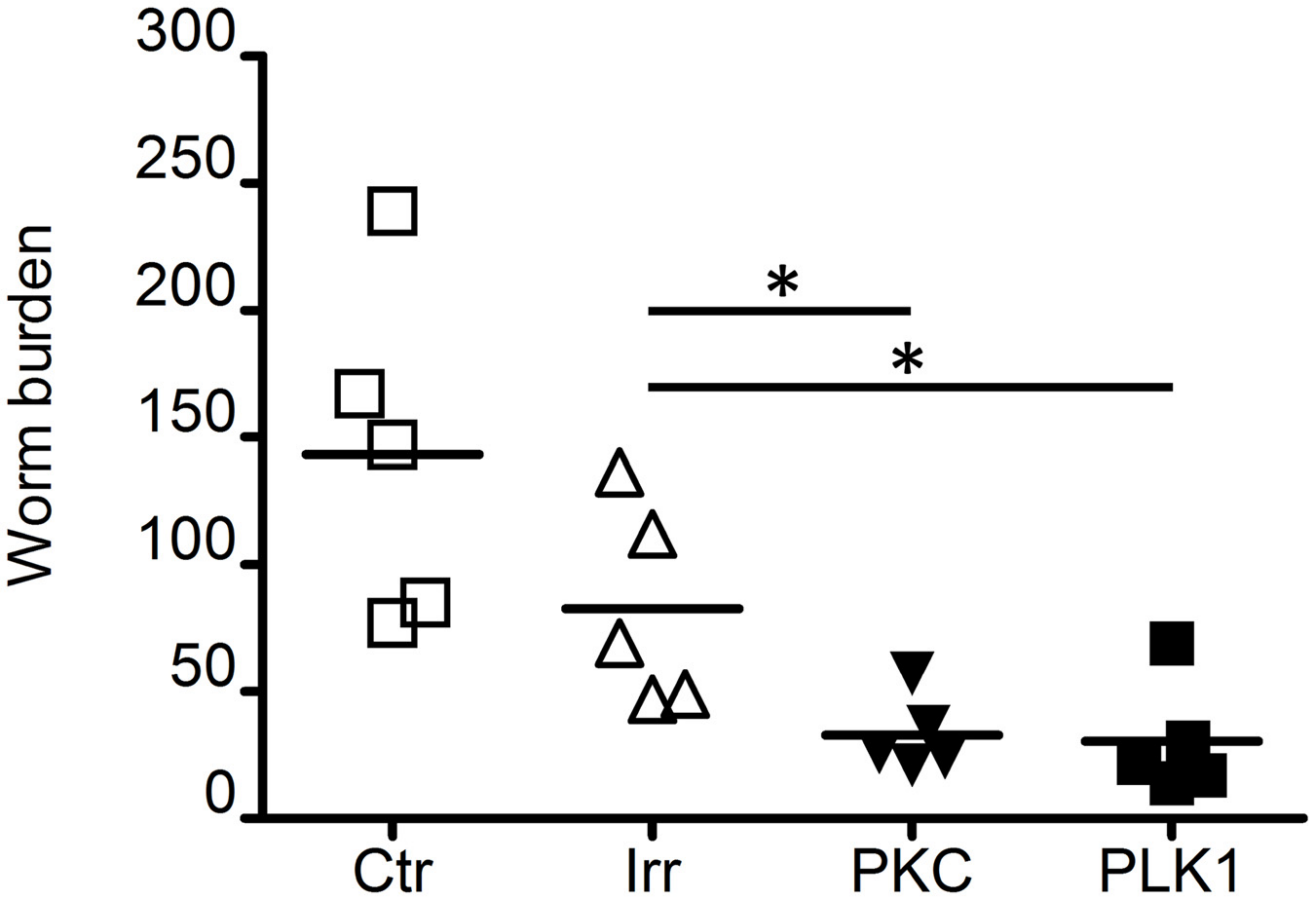
Worm recovery following *in vivo* injection of schistosomula silenced for *Sm-*aPKC or *Sm-*PLK1. Worms recovered by portal perfusion 4 weeks after i.m. injection of 1000 schistosomula treated with *Sm*-aPKC (aPKC), *Sm*-PLK1 (PLK1), irrelevant long dsRNA (IRR) or untreated (CTR). Significant differences were assessed by unpaired Student’s t-test; * = P<0.05.

Groups of 5 mice were injected intramuscularly with 1,000 control, *Sm*-aPKC or *Sm*-PLK1 treated schistosomula/mouse and perfused 4 weeks later. Aliquots of the same batches of schistosomula were cultured *in vitro*. Data representative of two independent experiments is shown in Fig 8.

Mice injected with both *Sm-*aPKC and *Sm*-PLK1 silenced schistosomula yielded significantly fewer parasites compared with untreated controls or mice injected with IRR dsRNA. However, none of the surviving suppressed parasites showed any differences in size or shape compared to the controls. The recovered worms also showed no persisting gene suppression in contrast to the *Sm-*aPKC or *Sm*-PLK1 treated larvae maintained in culture for 4 weeks which showed significant knockdown (~70% and 80% respectively) comparable to that assessed one week after treatment.

## Discussion

In this study we set out to contribute to RNAi based validation of putative drug targets for schistosomiasis. *S*. *mansoni* possesses a quite large (270 Mb) and complex genome containing between 15,000 and 25,000 genes [22, 72]. It is thus crucial to find new *in silico* strategies to select genes which have a potential to be target candidates for new drugs or vaccines.

In looking for essential genes in pathogens a variety of characteristics can be considered. Crowther et al., [20] used the targets database (TDR) (http://tdrtargets.org/) as a tool to illustrate how to prioritize proteins which have a potential to be considered drug targets in neglected-disease causing pathogens. Such target selection has been applied to several parasitic diseases such as Chagas disease, parasitic worm infections and malaria. The strategy is based on a multi-criteria search through the use of the open-access resource in TDR in which it is possible to manually select proteins that fit specific desired traits (druggability, assayability, essentiality). In *S*. *mansoni* proteins are not yet scored for druggability in the TDR database, so their druggability was assessed by comparing the proteins’ amino acid sequences to those of known targets in the protChEMBL database. In addition to this, proteins’ functional importance have been compared to gene knockout data of their orthologs in *C*. *elegans* and *D*. *melanogaster*. This method yielded a top list of 170 *Schistosoma* targets.

Caffrey et al. [68], focused their search on a comparative chemogenomics approach based on the proteome of two model organisms, the nematode *Caenorhabditis elegans* and the fruit fly, *Drosophila melanogaster* in which gene knockout studies had identified proteins which appear to be essential or producing a deleterious phenotype in both organisms. The search aimed to highlight proteins in these organisms having clear sequence similarities to orthologues in schistosomes. This type of manual screening led to a selection of 35 *S*. *mansoni* druggable targets 8 of which were also present in the earlier list [20].

Our strategy was similar to the above and aimed at identifying potential essential and druggable targets for *S*. *mansoni* by inference from the ChEMBL database of drug targets and orthology essential data from *C*. *elegans*, as a one of the few model systems where whole genome functional genomics studies using RNAi have been performed. This analysis produced a list of 24 target candidate genes to be analysed by RNAi of which only the glycogen synthase kinase 3-related (gsk3) gene (Smp_008260) was also present in the earlier lists [68].

In the case of *Sm-*Calm, the first gene on our list of 24 genes, robust suppression was induced in adult worms and resulted in regular waves of contraction and dilation in male worms but not females. Interestingly, siRNA knockdown of a 24kDa calcium-regulated heat-stable protein of *S*. *japonicum* (CRHSP-24) induces what appears to be a similar contraction wave phenotype in juvenile *S*. *japonicum* worms [73]. Calmodulin as a transporter of Ca^2+^ plays a key role in calcium signalling, affecting numerous functions including muscle contraction and metabolism. Selective inhibitors of calmodulin in schistosomes block egg hatching [74] and both inhibitors [75] and siRNA knockdown [76] inhibit miracidial transformation to sporocysts. Exposure of worms to PZQ results in disruption of Ca^2+^ homeostasis mediating its action by inducing Ca^2+^ influx [5] and it has been suggested that molecules in the calcium signalling pathway may be potential targets for drug discovery. After 5 weeks the contraction phenotype waned but by this time the worms were slow, darkened and had tegumental damage. Despite the important role that calmodulin plays during different stages of *S*. *mansoni* development the very slow induction of damage to adults and the fact that the gene exhibits more than 90% identity with mammalian calmodulin [76] reduce its suitability as a target for drug development.

Silencing of the atypical protein kinase C (*Sm-*aPKC) similarly had no immediate effect on adult worms but after 2 weeks in culture the worms became darkened, non-adherent and developed blebs. By the third week they were severely damaged. *Sm-*aPKC silencing in schistosomula also induced decreased viability and development after 2 weeks of *in vitro* culture especially when cultured in the presence of hRBC. Reduced viability was also seen when *Sm-*aPKC silenced schistosomula were injected *in vivo* supporting a role for this gene in schistosomula development.

Interestingly we found that parasites recovered from mice *in vivo* showed no more silencing resulting in mRNA levels comparable to controls. Such phenomenon is not surprising as it has been previously observed in other studies [32, 42, 63].

As suggested by Krautz-Peterson et al., one hypothesis is that different schistosomula can receive variable amounts of dsRNA or, alternatively, this disparity could be due to a more active metabolism of parasites *in vivo* that can determine a shorter half life of the dsRNA [77].

Members of the family of atypical PKCs are considered to be key components of downstream signalling pathways activated by the EGF receptors which can activate diverse signal transduction cascades [78]. Earlier studies on another *S*. *mansoni* protein kinase C (*Sm*PKC1), with high homology to mammalian PKCβ, was found to be differentially expressed during the schistosome life cycle, with the highest mRNA and protein levels being found in miracidia and sporocysts, respectively. The protein was also immunolocalized to the acetabular glands of newly transformed schistosomula suggesting a possible role in larval transformation [48]. A further study demonstrated activated schistosome PKCβ expression during postembryonic development of the miracidium to mother sporocyst suggesting its role in regulating this transformation [79]. The effect that *Sm-*aPKC silencing had on both adult and larvae schistosomes in our studies indicates an important role in the parasite although the slow onset of effects does not support it as a likely drug target.

Given the importance of kinases as potential drug targets and having shown some phenotypic effects following knockdown of the atypical protein kinase C (*Sm-*aPKC) we focused our attention on additional kinases. Andrade et al., [28] identified and classified 252 *S*. *mansoni* eukaryotic protein kinases (ePKs), describing some of them as good drug candidates as they may perform an essential function in the parasite. Although they often had high sequence similarity to their human homologues, new and effective drugs can overcome such similarity by binding protein kinases close but not in the ATP site occluding ATP access to the kinase to retard enzyme activity [28]. Initially we carried out RNAi suppression on four other kinases in our list of putative essential genes alongside three other non-kinase genes but failed to demonstrate any phenotype with these knockdowns. So attention was switched to selecting genes through computational prediction of putative targets based on either searching the ChEMBL database for compounds found to be active against adult *S*. *mansoni* in previous whole organism screens [16, 45, 80] or from the focused PKIS kinase inhibitor collection and the kinase inhibitors represented in the DDU compound collection we had tested previously [45]. A similar study was recently conducted in zebrafish by Laggner *et al*. that sought to identify putative targets of 681 neuroactive molecules from a 14,000-compound phenotypic screen in zebrafish embryos [81]. Putatively homologous genes for the identified kinases were mapped in the *S*. *mansoni* genome and thereafter 10 kinases highlighted to be validated by RNAi phenotypic screening.

From this screening we obtained detectable phenotype in schistosomula but not in adults following the knockdown of 2 of the 10 genes tested, *Sm-*PLK1 and *Sm*-MAPK p38. As for *Sm*-aPKC the loss in schistosomula viability with *Sm*-PLK-1 and *Sm*-MAPK p38 started after 2–3 weeks and were more marked in cultures with hRBC in which the larvae were developing rapidly indicating a key role for these kinases during development. In the case of PLK-1 the RNAi effects were exactly comparable when the PLK-1 specific inhibitor BI2536 was used.

The failure to demonstrate activity in the adults with the RNAi knockdowns despite the adult stage having been used initially to screen the kinase inhibitors may be explained by the relatively modest effects seen against adults in the initial *in vitro* testing i.e. the highest percentage inhibition of adult motility achieved with the five GSK kinase inhibitors identified to target PLK-1 was 65% and the next highest only 25%. P38 MAPK was implicated by two inhibitors inducing 82% and 76% inhibition of motility. So none of these inhibitors actually killed the adults which combined with the only partial, albeit high, RNAi knockdown may have explained why no effect on phenotype was seen with the RNAi in adults. By comparison the phenotypic effects seen in schistosomula may reflect greater sensitivity of this developing stage to knockdown of these two genes. It also cannot be ruled out that the compounds inhibit additional targets (possibly protein kinases) within the schistosomes, complicating the analysis.

PLKs constitute a family of serine threonine kinases which have a highly conserved function in all eukaryotes. Along with other mitotic kinases, they play a crucial role in coordinating cell cycle progression and are potent regulators of M phase [82]. In vertebrates, there are four well-known PLKs (PLK 1–4) whilst schistosomes only possess two, *Sm-*PLK1 and *Sm-*Sak, both well characterized [71, 83, 84]. *Sm-*PLK1 has been shown to be abundantly expressed in germinal and vitelline cells and more abundant in sporocysts than in other parasite stages, consistent with its potential role in mitosis and/or meiosis in schistosomes. Our data suggest that *Sm-*PLK1 might play an important role also at the schistosomula stage which is characterized by rapid growth and body remodeling particularly when hRBC are added, processes which would require active mitotic machinery. Such an important role in schistosomula can also be observed *in vivo* where a reduced viability was also seen in silenced parasites injected in mice thus supporting the involvement for this gene in schistosomula development.

In addition, previous studies have shown that adult worms treated with BI2536, a specific inhibitor of *Sm-*PLK1, which showed altered morphology of reproductive organs with significant effects on oogenesis and spermatogenesis [83]. In our studies neither RNAi knockdown or BI2536 affected adult worm viability but BI2536 inhibited embryonation of eggs produced *in vitro* as would be expected from inhibition of mitosis.

Similar effects in terms of morphology and kinetics were seen with silencing of the *Sm*-MAPK p38 family member (Fig 7). It has been previously shown how p38 MAPK inhibition with pyridinylimidazole can have crucial effects on survival and replication of some pathogens such as *P*. *falciparum* and *T*. *gondii* [85, 86]. The active form of this gene in *S*. *mansoni* has been associated with the cilia of miracidia, playing a role in the ciliary motion [87] and also during the early stages of post-embryonic development [88]. Treatment performed with a specific inhibitor (SB203580) is able to retard the development of the miracidium to post-miracidium stage and also from post-miracidium to mother sporocyst stage, without affecting larval viability [88]. We demonstrated here that *Sm*-MAPK p38 might also play an important role in schistosomula development and that its suppression affects growth and survival of parasites *in vitro*.

Despite the coincidence of the nature and timing of the effects seen *in vitro* with these different RNAi gene knockdowns in schistosomula we believe the effects to be specific for the following reasons: (i) IRR dsRNA prepared in exactly same way as that for the specific genes was used in all experiments and cause no significant phenotypes; (ii) the effect was reproducible for the genes in question whereas no effects were seen for a number of other genes in which the RNAi was prepared in exactly the same way; (iii) in a recent study of RNAi knock-down of 3-hydroxy-3-methyl-glutaryl-CoA reductase (HMGR), the target for statins, there was, similarly little effect on schistosomula viability until 2–3 weeks of culture [36]; (iv) in the case of the *Sm-*PLK-1 knockdown the phenotype and time of its onset were exactly replicated with the PLK-1 inhibitor, BI2356; (v) in the cultures with hRBC the loss of viability is seen once larval development characterized by gut formation is initiated whereas schistosomula which retain a “lung-form” morphology remain unaffected.

Although we were able to demonstrate phenotypic effects on both larval and adult schistosomes, the earliest effects seen were at 2 weeks (calmodulin in adults) whilst most of the effects on larvae were not seen until the second/third week. Furthermore, with some of the gene knockdowns effects were seen with larvae but not adults and vice versa. In addition the deleterious effects on schistosomula were markedly enhanced and visualized when hRBC were added and the larvae induced to feed. Indeed it was commonly seen that larvae which did not feed and retained the lung form phenotype seemed unaffected (e.g. Fig 5E). It is not surprising that the induction of development following initiation of red blood cells is associated with expression of genes vital for survival. Microarray studies on schistosomula cultured for 5 days have characterized the transcriptional changes in genes encoding surface and secreted proteins during the first 5 days in *in vitro* culture and show upregulation of a number of genes in the presence of hRBC [70].

We conclude that RNAi of schistosomula combined with culture in the presence of hRBC is a valuable approach to understanding key roles played by individual genes. However, as mentioned at the outset one of our intentions was to assess whether the automated image analysis of larval motility and morphology which we have developed for drug screening [45] could be developed for HTS RNAi. Effects which take weeks to manifest, vary markedly between individual worms, and rely on addition of hRBC are not conducive to this sort of analysis not least because once hRBC are added to schisostomula, the individual worms develop at different rates some developing guts quickly and others remaining like lung forms. So there is even more variation in shape, size, motility and appearance than with newly transformed parasites making development of segmentation and image analysis of morphological changes more challenging. Nevertheless, inhibition of motility would be easier to develop although the presence of hRBC would interfere with segmentation of the parasites for image analysis. This proof of concept study illustrates the potential of combining bioinformatics target prioritization with RNAi viability studies as a method to identify putative drug targets in *S*. *mansoni*.

## Supporting Information

S1 Table T7 promoter-tagged primers. (DOCX)

S2 Table Putatively essential genes selected for *S*.*mansoni*. (DOCX)

S3 Table GlaxoSmithKline Published Kinase Inhibitor Set (PKIS). (DOCX)

S4 Table Silencing results and phenotypic effect in adult and larval stages following RNAi of genes selected from GSK and DDU library. (DOCX)

S5 Table Silencing results and phenotypic effects in adult and larval stages following RNAi of putatively essential genes. (DOCX)

S1 Fig DDU library target assignment via ChEMBL prediction similarity network. Compounds similarity network for the Dundee set (singletons not displayed). Compounds are coloured with blue = non hit and red = hit.
(TIFF)

S2 Fig GSK library targets assignment in *S*. *mansoni* similarity network. Compounds similarity network of the GSK set (singletons not displayed). Protein targets identified in *S*. *mansoni* based on the annotation from GSK. Colours are based on the % motility reduction going from 0% (blue) and from 1 to 100% (yellow to red gradient). Some cluster examples are illustrated with proteins names above them (PLK1 is Serine/threonine-protein kinase PLK1, p38 is MAP kinase p38, VEGFR2 is Vascular endothelial growth factor receptor 2, EGFR/ErbB2 is Epidermal growth factor receptor and Receptor protein-tyrosine kinase erbB-2, GSK3 is Glycogen synthase kinase-3, IGF1R is Insulin-like growth factor I receptor).
(TIF)

S3 Fig Suppression of *Sm*-Calm using both siRNA and long dsRNA. Relative expression of *Sm*-Calm in adult parasites electroporated with either 2.5μg/ml of synthetic siRNA (1 and 2 from Applied Biosystem, 3 and 4 from IDT) or 100μg/ml of long dsRNA and their respective IRR controls.
(TIFF)
