# Supplementary material for: Application of RNAi to Genomic Drug Target Validation in Schistosomes
Source: PLoS Negl Trop Dis. 2015 May 20;9(5):e0003801. doi: 10.1371/journal.pntd.0003801 (PMC4438872; doi:10.1371/journal.pntd.0003801)
Supplement: S1 Table — (DOCX) [file pntd.0003801.s001.docx]

| Gene | Description | Forward primer (5’ T7 tail) | Reverse primer (5’ T7 tail) |
| --- | --- | --- | --- |
| Smp_026560.2 | Calmodulin, putative | GCCGACCAATTAACAGAGGA | TTTCATCGACCTCGTCATCA |
| Smp_096310 | Serine/threonine kinase | CACGATTCTATGCAGCTGAG | ACTTGAATTTGGCGCACATC |
| Smp_008260 | Glycogen synthase kinase 3-related (gsk3) (cmgc group III) | GGATCATTTGGCGTTGTG | GCACCAAATATCAATTCAGGA |
| Smp_141380 | Serine/threonine kinase | TGGAGTCGGATGAAACAAGT | TCACTGTCTGAAAACAAATCAATAAG |
| Smp_180400 | Serine/threonine kinase | TGATTGGGAATTCGATTGGTG | TCCGACACCAGTAGGTCCA |
| Smp_080730 | Serine/threonine kinase | ACTTGCAGATTTCGGTTTGG | ATATACTTCGCCGCCATCTG |
| Smp_093930.2 | Receptor Tyrosine Kinase, putative | TGTCGTGTTTGGGAGAGAGA | GGACAGGAACCATGACAGG |
| Smp_165470 | Tyrosine Kinase Receptor, EGFR family | TGACTACACAATGTCAGCATCC | CAGCAGCCTCGAATGATGTA |
| Smp_152680 | Tyrosine Kinase Receptor, EGFR family | TCCATGTTGTGTAAGATTTTTAGCAT | TTTCCGGTTGAGGTAAACGA |
| Smp_140700 | Serine/Threonine Kinase, MAPK family | CAATCCTCCAGCTCATTTCG | TGACAAAAATAAGGGGATACGC |
| Smp_133020 | Serine/Threonine Kinase, MAPK family, p38 subfamily | CCGTCGTGTAGCCATTAAAAAG | TGGATCATCGAATCTCTCTGC |
| Smp_191040 | P38 MAPK, putative | AGTATTGGCTTAATTCATCGA | GAATAAATATACATTTTTTATTACCAG |
| Smp_009990 | Tyrosine Kinase Receptor, InsR family | GATAACAATTACAAATGGAGATTTAGC | GTGAATTTTTATTGACAGGTTC |
| Smp_009600 | Hybrid Protein Kinase, PLK1 subfamily | GTGAAGTTCCAGCAGCCT | GTGCGTATCTCAATCTGTTTG |
| Smp_151300 | Proto-oncogene tyrosine-protein kinase src, putative | CAGATACGCTACCAACCCTGTA | CTGATAAAGAATATTCACTGGATTG |
| Smp_157300 | Tyrosine Kinase Receptor | GTTTTCCGATTCAACTATAACGATG | GTAATAATTTCGGATTTTCGTCTC |
