# Supplementary material for: Application of RNAi to Genomic Drug Target Validation in Schistosomes
Source: PLoS Negl Trop Dis. 2015 May 20;9(5):e0003801. doi: 10.1371/journal.pntd.0003801 (PMC4438872; doi:10.1371/journal.pntd.0003801)
Supplement: S2 Table — (DOCX) [file pntd.0003801.s002.docx]

| **Gene** | **Description** | **Startlite**  **link** | **Startlite Description** | **Organism** |
| --- | --- | --- | --- | --- |
| Smp_026560.2 | calmodulin, putative | [10173](http://www.ebi.ac.uk/chembldb/index.php/target/inspect/10173) | Calcineurin B subunit isoform 1 | *Homo sapiens* |
| Smp_096310 | serine/threonine kinase | [10901](http://www.ebi.ac.uk/chembldb/index.php/target/inspect/10901) | Protein kinase C, iota type (nPKC-iota) | *Homo sapiens* |
| Smp_194160 | leucyl-tRNA synthetase, putative | [12060](http://www.ebi.ac.uk/chembldb/index.php/target/inspect/12060) | Leucyl-tRNA synthetase, cytoplasmic (Leucine--tRNA ligase) | *Homo sapiens* |
| Smp_089700 | integrin beta subunit, putative | [12662](http://www.ebi.ac.uk/chembldb/index.php/target/inspect/12662) | Integrin beta-2 precursor (Cell surface adhesion glycoproteins LFA-1/CR3/p150,95 beta-subunit) | *Homo sapiens* |
| Smp_091770 | protein farnesyltransferase alpha subunit, putative | [12228](http://www.ebi.ac.uk/chembldb/index.php/target/inspect/12228) | Protein farnesyltransferase/geranylgeranyltransferase type I alpha subunit | *Bos taurus* |
| Smp_085740 | abl-bindingprotein-related | [12179](http://www.ebi.ac.uk/chembldb/index.php/target/inspect/12179) | growth factor receptor-bound protein 2 isoform 2 | *Homo sapiens* |
| Smp_009030 | ribonucleoside-diphosphate reductase, alpha subunit, putative | [98](http://www.ebi.ac.uk/chembldb/index.php/target/inspect/98) | Ribonucleoside-diphosphate reductase large subunit (Ribonucleoside-diphosphate reductase M1 subunit | *Homo sapiens* |
| Smp_034670 | tubulin gamma chain, putative | [33](http://www.ebi.ac.uk/chembldb/index.php/target/inspect/33) | Tubulin beta-2 chain | *Homo sapiens* |
| Smp_041600 | isoleucine--tRNA ligase | [11787](http://www.ebi.ac.uk/chembldb/index.php/target/inspect/11787) | Isoleucyl-tRNA synthetase, cytoplasmic (Isoleucine--tRNA ligase) (IleRS) (IRS) | *Homo sapiens* |
| Smp_008260 | glycogen synthase kinase 3-related (gsk3) (cmgc group III) | [10197](http://www.ebi.ac.uk/chembldb/index.php/target/inspect/10197) | Glycogen synthase kinase-3 beta (GSK-3 beta) | *Homo sapiens* |
| Smp_141380 | protein kinase | [20072](http://www.ebi.ac.uk/chembldb/index.php/target/inspect/20072) | Cell division control protein 2 homolog (p34 protein kinase) | *Mus musculus* |
| Smp_180400 | serine/threonine kinase | [20072](http://www.ebi.ac.uk/chembldb/index.php/target/inspect/20072) | Cell division control protein 2 homolog (p34 protein kinase) | *Mus musculus* |
| Smp_073410 | proteasome catalytic subunit 2 (T01 family) | [11409](http://www.ebi.ac.uk/chembldb/index.php/target/inspect/11409) | Dual specificity mitogen-activated protein kinase kinase 1 (MAP kinase kinase 1) | *Homo sapiens* |
| Smp_076230 | proteasome subunit alpha 7 (T01 family) | [100624](http://www.ebi.ac.uk/chembldb/index.php/target/inspect/100624) | Proteasome subunit beta type 5 precursor | *Homo sapiens* |
| Smp_170730 | proteasome subunit alpha 1 (T01 family) | [100624](http://www.ebi.ac.uk/chembldb/index.php/target/inspect/100624) | Proteasome subunit beta type 5 precursor | *Homo sapiens* |
| Smp_055890 | ribonucleoside-diphosphate reductase small chain, putative | [11257](http://www.ebi.ac.uk/chembldb/index.php/target/inspect/11257) | Ribonucleoside-diphosphate reductase M2 chain (Ribonucleotide reductase small chain) | *Homo sapiens* |
| Smp_173810 | protein phosphatase pp2a regulatory subunit B, putative | [17130](http://www.ebi.ac.uk/chembldb/index.php/target/inspect/17130) | Serine-threonine protein phosphatase 2A regulatory subunit | *Gallus gallus* |
| Smp_027880 | expressed protein | [10342](http://www.ebi.ac.uk/chembldb/index.php/target/inspect/10342) | MHC class II region expressed gene KE2 | *Rattus norvegicus* |
| Smp_165490 | protein phsophatase-2a, putative | [12578](http://www.ebi.ac.uk/chembldb/index.php/target/inspect/12578) | Serine/threonine protein phosphatase 2A, catalytic subunit, alpha isoform (PP2A-alpha) | *Bos taurus* |
| Smp_035580 | protein phosphatase-1, putative | [12999](http://www.ebi.ac.uk/chembldb/index.php/target/inspect/12999) | Serine/threonine protein phosphatase PP1-gamma catalytic subunit (PP-1G) | *Homo sapiens* |
| Smp_040770 | methionine-tRNA synthetase, putative | [12818](http://www.ebi.ac.uk/chembldb/index.php/target/inspect/12818) | Methionyl-tRNA synthetase | *Homo sapiens* |
| Smp_164840 | proteasome catalytic subunit 3 (T01 family) | [100624](http://www.ebi.ac.uk/chembldb/index.php/target/inspect/100624) | Proteasome subunit beta type 5 precursor | *Homo sapiens* |
| Smp_080730 | serine/threonine kinase | [20072](http://www.ebi.ac.uk/chembldb/index.php/target/inspect/20072) | Cell division control protein 2 homolog (p34 protein kinase) | *Mus musculus* |
| Smp_157090 | subfamily C1A unassigned peptidase (C01 family) | [10498](http://www.ebi.ac.uk/chembldb/index.php/target/inspect/10498) | Cathepsin L precursor | *Homo sapiens* |
