# Supplementary material for: Application of RNAi to Genomic Drug Target Validation in Schistosomes
Source: PLoS Negl Trop Dis. 2015 May 20;9(5):e0003801. doi: 10.1371/journal.pntd.0003801 (PMC4438872; doi:10.1371/journal.pntd.0003801)
Supplement: S3 Table — (DOCX) [file pntd.0003801.s003.docx]

| **ID Number** | **% Motility Reduction** | **Additional comments** | **SMILES** | **Ref** |
| --- | --- | --- | --- | --- |
| **HIT: minimal motility/ severe tegumental damage, dark, granular, opaque** | | | | |
| GSK1173862A | 100 |  | CCCN1CCCC(C1)c1ccc(Nc2nc(Nc3cc(F)ccc3C(N)=O)c3cc[nH]c3n2)c(OC)c1 | [[1](#_ENREF_1)] |
| SB-278538 | 81.8 | ♀>♂ | CC(C)(C)c1ccc(Oc2nccc(n2)-c2c(ncn2C2CCNCC2)-c2ccc(F)cc2)cc1 | [[2](#_ENREF_2)] |
| SB-245392 | 76 | ♀>♂ | Fc1ccc(cc1)-c1ncn(C2CCNCC2)c1-c1ccnc(Oc2ccc(Oc3ccccc3)cc2)n1 | [[2](#_ENREF_2)] |
| SB-400868-A | 71 | ♀>♂ | Cl.C1Cc2nc(c(-c3ccc4OCOc4c3)n2C1)-c1ccccn1 | [[3](#_ENREF_3)] |
| GW795486X | 70 |  | Nc1ncnc2occ(-c3ccc(NC(=O)Nc4cc(cc(c4)C(F)(F)F)C(F)(F)F)cc3)c12 | [[4](#_ENREF_4)] |
| **Slowed motility/ tegumental damage, dark granular, opaque** | | | | |
| GSK2186269A | 67.5 | ♂>♀ | COc1cc2CCN(C(=O)CN(C)C)c2cc1Nc1nc(N2CCc3ccccc23)c2cc[nH]c2n1 | [[5](#_ENREF_5)] |
| GSK571989A | 65 | ♀>♂ | C[C@@H](Oc1cc(sc1C(N)=O)-n1cnc2ccc(OCC3CCN(C)CC3)cc12)c1ccccc1Cl | [[6](#_ENREF_6)] |
| **Slowed motility/dark granular, opaque** | | | | |
| GW795493X | 62.5 | Elongated | Nc1ncnc2occ(-c3ccc(NC(=O)Nc4cccc(c4)C(F)(F)F)cc3)c12 | [[4](#_ENREF_4)] |
| GW772405X | 50 |  | CNC(=O)c1cccc(c1)C#Cc1cncnc1Nc1ccc(OCc2cccc(F)c2)c(Cl)c1 | [[7](#_ENREF_7)] |
| GW817394X | 50 |  | COc1cccc(c1)-n1ncc2c(N\N=C\c3cccc(F)c3)ncnc12 | [[8](#_ENREF_8)] |
| GW770249A | 25 | ♂ enlarged | Cl.Nc1ncnc2occ(-c3ccc(NC(=O)Nc4cc(ccc4F)C(F)(F)F)cc3)c12 | [[4](#_ENREF_4)] |
| GSK2220400A | 25 | ♂ enlarged | CNC(=O)c1ncccc1Nc1nc(Nc2cc3N(CCCc3cc2OC)C(=O)CN(C)C)nc2[nH]ccc12 | [[5](#_ENREF_5)] |
| GSK2163632A | 25 | ♂ enlarged | COc1cc2c(cc1Nc1nc(Nc3ccsc3C(N)=O)c3cc[nH]c3n1)N(CCC2(C)C)C(=O)CN(C)C | [[5](#_ENREF_5)] |
| GW770249X | 25 | Elongated, slender | Nc1ncnc2occ(-c3ccc(NC(=O)Nc4cc(ccc4F)C(F)(F)F)cc3)c12 | [[4](#_ENREF_4)] |
| GW684626B | 25 | Shortened | Fc1cccc(COc2ccc(Nc3ncnc4sc(cc34)-c3cccs3)cc2Cl)c1 | [[9](#_ENREF_9)] |
| GW621970X | 25 | Shortened | CCS(=O)(=O)c1ccc(OC)c(Nc2ncc(o2)-c2cccc(F)c2)c1 | [[10](#_ENREF_10)] |
| GW843682X | 25 | Shortened | COc1cc2ncn(-c3cc(OCc4ccccc4C(F)(F)F)c(s3)C(N)=O)c2cc1OC | [[6](#_ENREF_6)] |
| GW620972X | 25 | Shortened | O=C(Nc1sc2CCCCc2c1C#N)c1cccc2ccccc12 | [[11](#_ENREF_11)] |
| GSK2213727A | 25 | Shortened | COc1cc(C)c(NC(=O)CN(C)C)cc1Nc1nc(Nc2cccc(F)c2C(N)=O)c2cc[nH]c2n1 | [[5](#_ENREF_5)] |
| GW807930X | 25 | Tegument blebbing | CC(=O)NCc1cccc(c1)C#Cc1cncnc1Nc1ccc(OCc2cccc(F)c2)c(Cl)c1 | [[7](#_ENREF_7)] |
| GW440139A | 25 | Tegument blebbing | Cl.Cc1ccc(O)cc1Nc1ccnc2cc(ccc12)-c1ccccn1 | [[12](#_ENREF_12)] |
| SB-725317 | 25 |  | Oc1ccc(cc1)-c1nc2[nH]nc(NC(=O)C3CC3)c2cc1Br | [[13](#_ENREF_13)] |
| GW284372X | 25 |  | C(Oc1ccc(Nc2ncnc3ccc(cc23)-c2ccco2)cc1)c1ccccc1 | [[14](#_ENREF_14)] |
| GW779439X | 25 |  | CN1CCN(CC1)c1ccc(Nc2nccc(n2)-c2cnn3ncccc23)cc1C(F)(F)F | [[15](#_ENREF_15)] |
| GW768505A | 25 |  | Cl.COc1ccc(cc1)-c1oc2ncnc(N)c2c1-c1ccc(NC(=O)Nc2cc(ccc2F)C(F)(F)F)cc1 | [[4](#_ENREF_4)] |
| GSK1713088A | 25 |  | CCCN1CCC(CC1)Oc1cc(Nc2nc(Nc3cccc(F)c3C(N)=O)c3cc[nH]c3n2)c(OC)cc1Cl | [[5](#_ENREF_5)] |
| GW703087X | 25 |  | CC(=O)Nc1cccc(c1)C#Cc1cncnc1Nc1ccc(OCc2cccc(F)c2)c(Cl)c1 | [[7](#_ENREF_7)] |
| GW780056X | 25 |  | CCN(CC)Cc1ccc(Nc2nccc(n2)-c2cnn3ncccc23)cc1 | [[15](#_ENREF_15)] |
| GW794726X | 25 |  | CC(=O)Nc1cccc(n1)C#Cc1cncnc1Nc1ccc(OCc2cccc(F)c2)c(Cl)c1 | [[7](#_ENREF_7)] |
| GW568377A | 25 |  | Cl.CS(=O)(=O)CCNCc1ccoc1-c1ccc2ncnc(Nc3ccc(OCc4ccccc4)cc3)c2c1 | [[14](#_ENREF_14)] |
| GW810576X | 25 |  | COc1cccc(Nc2nccc(n2)-c2cnn3ncccc23)c1 | [[16](#_ENREF_16)] |
| GW683134A | 25 |  | Cl.Fc1ccc(cc1NC(=O)Nc1ccc(Oc2ccc3[nH]c(NC(=O)c4ccco4)nc3c2)cc1)C(F)(F)F | [[17](#_ENREF_17)] |
| GSK319347A | 14 | ♀>♂: very damaged tegument, shortened | COc1cc2ncn(-c3cc(OCc4ccccc4S(C)(=O)=O)c(s3)C#N)c2cc1OC | [[18](#_ENREF_18)] |
| GW576924A | 12.5 | ♂>♀ | Cl.Fc1cc(Nc2ncnc3ccc(cc23)-c2ccc(CN3CCS(=O)CC3)o2)ccc1OCc1ccccc1 | [[14](#_ENREF_14)] |
| GW801372X | 12.5 | ♂>♀ | COc1cc(Nc2nccc(n2)-c2cnn3ncccc23)cc(OC)c1 | [[16](#_ENREF_16)] |
| GSK237701A | 12.5 | ♂: waves of body contraction.: ♀s hyperactive | COc1cc2ncn(-c3cc(O[C@H](C)c4ccccc4Cl)c(s3)C(N)=O)c2cc1OC | [[6](#_ENREF_6)] |
| GW569530A | 12.5 | ♂>♀, Tegument blebbing | Cl.CS(=O)(=O)CCNCc1nc(cs1)-c1ccc2ncnc(Nc3ccc(OCc4cccc(c4)C(F)(F)F)cc3)c2c1 | [[19](#_ENREF_19)] |
| GSK579289A | 12.5 | ♀>♂, elongated. | C[C@@H](Oc1cc(sc1C(N)=O)-n1cnc2ccc(OC3CCN(C)CC3)cc12)c1ccccc1Cl | [[6](#_ENREF_6)] |
| GW694234A | 8.9 | ♂ very shortened | Cl.COC(=O)Nc1nc2cc(Oc3ccc(NC(=O)Nc4cccc(Br)c4)cc3)ccc2[nH]1 | [[17](#_ENREF_17)] |
| SB-732881 | 8.3 | ♂>♀. Females: blebs at posterior ends | OC(=O)\C=C/C(O)=O.CN1CCC(CC1)C(=O)Nc1n[nH]c2nc(-c3ccc(O)cc3)c(Br)cc12 | [[20](#_ENREF_20)] |
| **Slow but not dark granular** | | | | |
| GW572401X | 25 |  | CCN(CC)S(=O)(=O)c1ccc(OC)c(Nc2ncc(o2)-c2ccccc2)c1 | [[10](#_ENREF_10)] |
| GW352430A | 25 |  | Cl.NS(=O)(=O)c1ccc(N\N=C2/C(=O)Nc3cccc(CCc4ccncc4)c23)cc1 | [[21](#_ENREF_21)] |
| GI261520A | 25 |  | Cl.COc1ccc2ncnc(Nc3ccc(OCc4ccccc4)cc3)c2c1 | [[22](#_ENREF_22)] |
| GW837331X | 25 |  | COc1cc(Nc2ncc3c(C)nc(-c4cccc(c4)C(F)(F)F)n3n2)cc(OC)c1OC | [[23](#_ENREF_23)] |
| GSK326090A | **6.25** | ♀: very slender, bulbous in parts. Some blebs | C[C@@H](Oc1cc(sc1C(N)=O)-n1cnc2ccc(OCC3CCN(C)CC3)cc12)c1ccccc1C(F)(F)F | [[6](#_ENREF_6)] |

| **Normal movement but dark and granular** | | | | | | | | | | |
| --- | --- | --- | --- | --- | --- | --- | --- | --- | --- | --- |
| GW709042A | GW589961A | SB-390527 | GW673715X | SB-682330-A | | | SB-333612 | GW832467X | GW794607X | GW607049C |
| GW641155A | GW694590A | GW853609X | SKF-86055 | GW578748X | | | GW778894X | SB-242719 | GW784684X | GSK2110236A |
| GW784307A | GW853606X | GW805758X | GW513184X | GW806290X | | | GW416981X | GW827106X | SB-253226 |  |
| **Normal movement, minor morphological abnormality** | | | | | | | | | | |
| GW396574X | GW442130X | GW804482X | GSK1030058A | | GW833373X | GW615311X | | GW693881A | GW693917A | GSK1030061A |
| GW814408X | GW806742X | GW827396X | GW833373X | | GW827099X | GW627512B | | GSK238583A | GSK300014A | GW632046X |
| **Unpaired** | | | | | | | | | | |
| GW622055X | GSK1220512A | GSK1326255A | SB-220025-R | | GW559768X | GSK625137A | | SB-732881-H | GW282449A |  |
| GSK1030059A | GW612286X | GW782907X | GW643971X | | GW861893X | GSK953913A | | GW809897X |  |  |

Compounds showing some phenotype against adult *S. mansoni* *in vitro* amongst the 367 GlaxoSmithKline Published Inhibitor Set (PKIS). For the 45 compounds showing most marked effects the SMILES string and the references pointing to the initial publication of the compound as a kinase inhibitor are shown above and below.

1. Chamberlain, S.D., et al., *Discovery of 4,6-bis-anilino-1H-pyrrolo[2,3-d]pyrimidines: potent inhibitors of the IGF-1R receptor tyrosine kinase.* Bioorg Med Chem Lett, 2009. **19**(2): p. 469-73.

2. Boehm, J.C., et al., *Phenoxypyrimidine inhibitors of p38alpha kinase: synthesis and statistical evaluation of the p38 inhibitory potencies of a series of 1-(piperidin-4-yl)-4-(4-fluorophenyl)-5-(2-phenoxypyrimidin-4-yl) imidazoles.* Bioorg Med Chem Lett, 2001. **11**(9): p. 1123-6.

3. Callahan, J.F., et al., *Identification of novel inhibitors of the transforming growth factor beta1 (TGF-beta1) type 1 receptor (ALK5).* J Med Chem, 2002. **45**(5): p. 999-1001.

4. Miyazaki, Y., et al., *Orally active 4-amino-5-diarylurea-furo[2,3-d]pyrimidine derivatives as anti-angiogenic agent inhibiting VEGFR2 and Tie-2.* Bioorg Med Chem Lett, 2007. **17**(6): p. 1773-8.

5. Chamberlain, S.D., et al., *Optimization of 4,6-bis-anilino-1H-pyrrolo[2,3-d]pyrimidine IGF-1R tyrosine kinase inhibitors towards JNK selectivity.* Bioorg Med Chem Lett, 2009. **19**(2): p. 360-4.

6. Emmitte, K.A., et al., *Design of potent thiophene inhibitors of polo-like kinase 1 with improved solubility and reduced protein binding.* Bioorg Med Chem Lett, 2009. **19**(6): p. 1694-7.

7. Waterson, A.G., et al., *Alkynyl pyrimidines as dual EGFR/ErbB2 kinase inhibitors.* Bioorg Med Chem Lett, 2006. **16**(9): p. 2419-22.

8. Peat, A.J., et al., *Novel pyrazolopyrimidine derivatives as GSK-3 inhibitors.* Bioorg Med Chem Lett, 2004. **14**(9): p. 2121-5.

9. Rheault, T.R., et al., *Thienopyrimidine-based dual EGFR/ErbB-2 inhibitors.* Bioorg Med Chem Lett, 2009. **19**(3): p. 817-20.

10. Harris, P.A., et al., *Discovery and evaluation of 2-anilino-5-aryloxazoles as a novel class of VEGFR2 kinase inhibitors.* J Med Chem, 2005. **48**(5): p. 1610-9.

11. Angell, R.M., et al., *N-(3-Cyano-4,5,6,7-tetrahydro-1-benzothien-2-yl)amides as potent, selective, inhibitors of JNK2 and JNK3.* Bioorg Med Chem Lett, 2007. **17**(5): p. 1296-301.

12. Graham Robinett, R., et al., *The discovery of substituted 4-(3-hydroxyanilino)-quinolines as potent RET kinase inhibitors.* Bioorg Med Chem Lett, 2007. **17**(21): p. 5886-93.

13. Witherington, J., et al., *6-heteroaryl-pyrazolo[3,4-b]pyridines: potent and selective inhibitors of glycogen synthase kinase-3 (GSK-3).* Bioorg Med Chem Lett, 2003. **13**(18): p. 3059-62.

14. Petrov, K.G., et al., *Optimization and SAR for dual ErbB-1/ErbB-2 tyrosine kinase inhibition in the 6-furanylquinazoline series.* Bioorg Med Chem Lett, 2006. **16**(17): p. 4686-91.

15. Stevens, K.L., et al., *Synthesis and evaluation of pyrazolo[1,5-b]pyridazines as selective cyclin dependent kinase inhibitors.* Bioorg Med Chem Lett, 2008. **18**(21): p. 5758-62.

16. Tavares, F.X., et al., *N-Phenyl-4-pyrazolo[1,5-b]pyridazin-3-ylpyrimidin-2-amines as potent and selective inhibitors of glycogen synthase kinase 3 with good cellular efficacy.* J Med Chem, 2004. **47**(19): p. 4716-30.

17. Hasegawa, M., et al., *Discovery of novel benzimidazoles as potent inhibitors of TIE-2 and VEGFR-2 tyrosine kinase receptors.* J Med Chem, 2007. **50**(18): p. 4453-70.

18. Bamborough, P., et al., *5-(1H-Benzimidazol-1-yl)-3-alkoxy-2-thiophenecarbonitriles as potent, selective, inhibitors of IKK-epsilon kinase.* Bioorg Med Chem Lett, 2006. **16**(24): p. 6236-40.

19. Gaul, M.D., et al., *Discovery and biological evaluation of potent dual ErbB-2/EGFR tyrosine kinase inhibitors: 6-thiazolylquinazolines.* Bioorg Med Chem Lett, 2003. **13**(4): p. 637-40.

20. Witherington, J., et al., *6-aryl-pyrazolo[3,4-b]pyridines: potent inhibitors of glycogen synthase kinase-3 (GSK-3).* Bioorg Med Chem Lett, 2003. **13**(18): p. 3055-7.

21. Bramson, H.N., et al., *Oxindole-based inhibitors of cyclin-dependent kinase 2 (CDK2): design, synthesis, enzymatic activities, and X-ray crystallographic analysis.* J Med Chem, 2001. **44**(25): p. 4339-58.

22. Zhang, Y.M., et al., *Synthesis and SAR of potent EGFR/erbB2 dual inhibitors.* Bioorg Med Chem Lett, 2004. **14**(1): p. 111-4.

23. Cheung, M., et al., *Imidazo[5,1-f][1,2,4]triazin-2-amines as novel inhibitors of polo-like kinase 1.* Bioorg Med Chem Lett, 2008. **18**(23): p. 6214-7.
