# Supplementary material for: Application of RNAi to Genomic Drug Target Validation in Schistosomes
Source: PLoS Negl Trop Dis. 2015 May 20;9(5):e0003801. doi: 10.1371/journal.pntd.0003801 (PMC4438872; doi:10.1371/journal.pntd.0003801)
Supplement: S4 Table — (DOCX) [file pntd.0003801.s004.docx]

| **Predicted target** | **Schisto gene** | **Description** | **Stage** | **% Silencing (range)** | **Phenotypic change** |
| --- | --- | --- | --- | --- | --- |
| EGFR  (P00533) | Smp_093930.2 | Receptor Tyrosine Kinase, putative | Larvae | 78-85 | - |
|  |  |  | Adults | 80-88 | - |
|  | Smp_165470 | Tyrosine Kinase Receptor, EGFR family | Larvae | 80-99 | - |
|  |  |  | Adults | 70-75 | - |
|  | Smp_152680 | Tyrosine Kinase Receptor, EGFR family | Larvae | ND | - |
|  |  |  | Adults | ND | - |
| MAP kinase 14  (Q16539) | Smp_140700 | Serine/Threonine Kinase, MAPK family | Larvae | 75-95 | - |
|  |  |  | Adults | 85-90 | - |
|  | Smp_133020 | Serine/Threonine Kinase, MAPK family, p38 subfamily | Larvae | 85-99 | + |
|  |  |  | Adults | 70-85 | - |
|  | Smp_191040 | P38 MAPK, putative | Larvae | - |  |
|  |  |  | Adults | 80-85 | - |
| Insulin receptor  (P08069) | Smp_009990 | Tyrosine Kinase Receptor, InsR family | Larvae | 60-90 | - |
|  |  |  | Adults | 70-90 | - |
| PLK1  (P53350) | Smp_009600 | Hybrid Protein Kinase, PLK1 subfamily | Larvae | 85-90 | + |
|  |  |  | Adults | 90-99 | - |
| VEGFR-2  (P35968) | Smp_151300 | Proto-oncogene tyrosine-protein kinase src, putative | Larvae | 65-75 | - |
|  |  |  | Adults | 75-85 | - |
|  | Smp_157300 | Tyrosine Kinase Receptor | Larvae | 80-98 | - |
|  |  |  | Adults | 80-98 | - |
| Tyrosine-protein kinase receptor  FLT3  (P36888) | Smp_157300 | Tyrosine Kinase Receptor | Larvae | 80-98 | - |
|  |  |  | Adults | 80-98 | - |
|  | Smp_151300 | Proto-oncogene tyrosine-protein kinase src, putative | Larvae | 65-75 | - |
|  |  |  | Adults | 75-85 | - |

ND: Not possible to determine by Real Time PCR
