# Supplementary material for: Application of RNAi to Genomic Drug Target Validation in Schistosomes
Source: PLoS Negl Trop Dis. 2015 May 20;9(5):e0003801. doi: 10.1371/journal.pntd.0003801 (PMC4438872; doi:10.1371/journal.pntd.0003801)
Supplement: S5 Table — (DOCX) [file pntd.0003801.s005.docx]

| Gene | Description | Stage | Silencing %(range) | Phenotypic change |
| --- | --- | --- | --- | --- |
|  |  |  |  |  |
| Smp_026560.2 | calmodulin, putative | Larvae | 80-90 | - |
|  |  | Adults | 85-95 | + |
| Smp_096310 | serine/threonine kinase | Larvae | 75-95 | + |
|  |  | Adults | 80-90 | + |
| Smp_008260 | glycogen synthase kinase 3-related (gsk3) (cmgc group III) | Larvae | 55-65 | - |
|  |  | Adults | 80-95 | - |
| Smp_141380 | serine/threonine kinase | Larvae | 20-50 | - |
|  |  | Adults | 75-80 | - |
| Smp_180400 | serine/threonine kinase | Larvae | 55-60 | - |
|  |  | Adults | 75-85 | - |
| Smp_080730 | serine/threonine kinase | Larvae | 80-90 | - |
|  |  | Adults | 80-85 | - |
