# Supplementary figures and images for: Application of RNAi to Genomic Drug Target Validation in Schistosomes
Source: PLoS Negl Trop Dis. 2015 May 20;9(5):e0003801. doi: 10.1371/journal.pntd.0003801 (PMC4438872; doi:10.1371/journal.pntd.0003801)

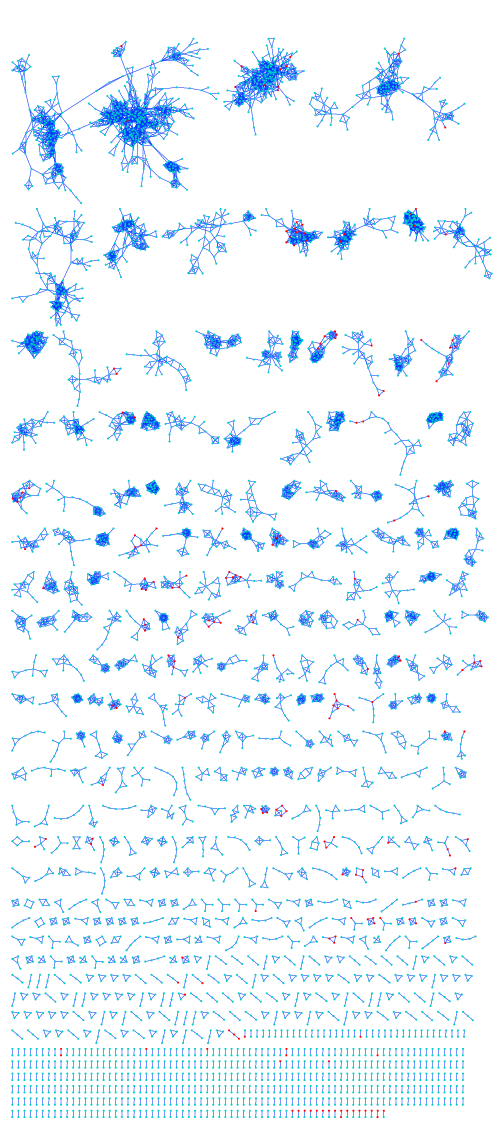

Supplement: S1 Fig — Compounds similarity network for the Dundee set (singletons not displayed). Compounds are coloured with blue = non hit and red = hit. (TIFF) [file pntd.0003801.s006.tiff]

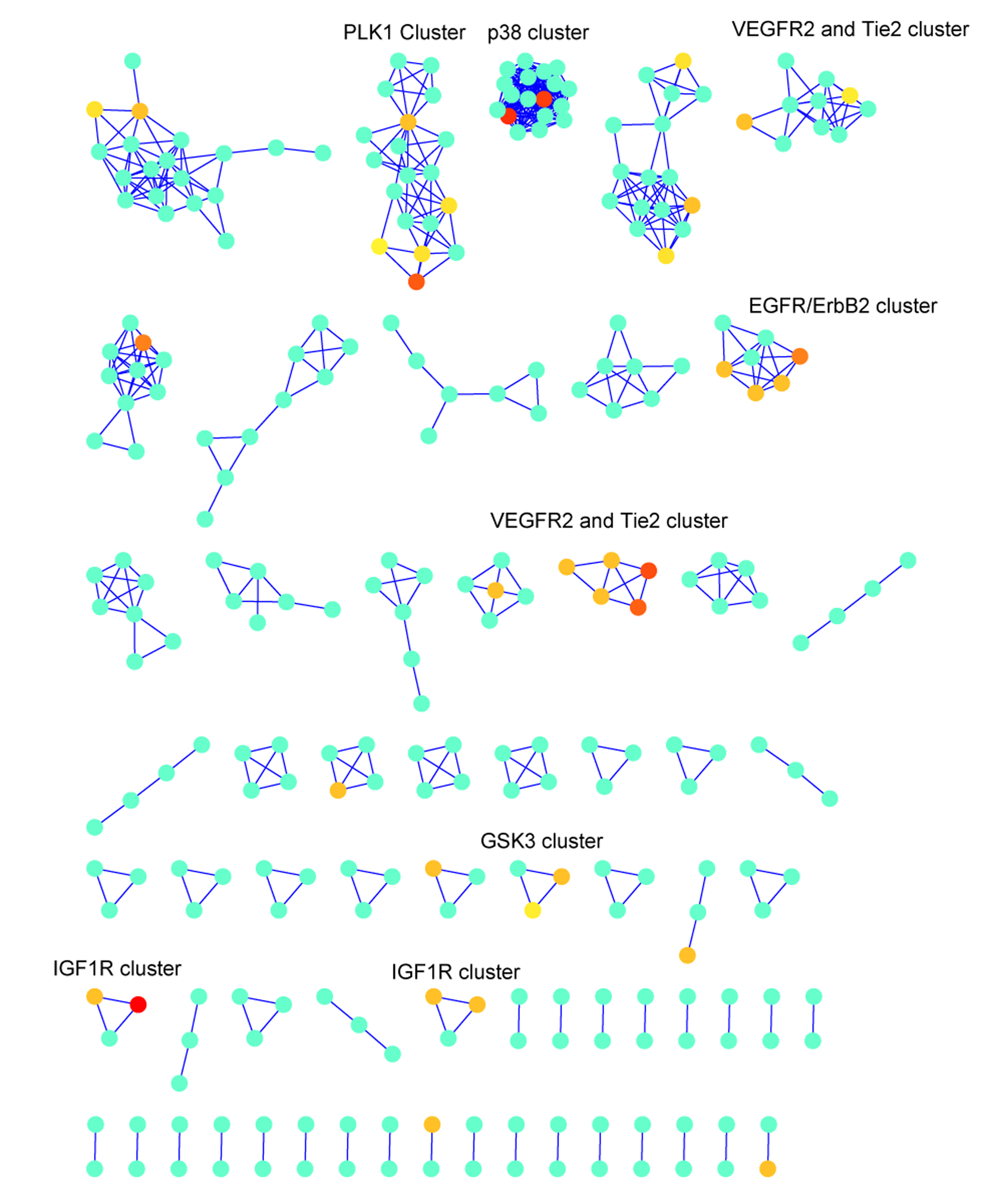

Supplement: S2 Fig — Compounds similarity network of the GSK set (singletons not displayed). Protein targets identified in S. mansoni based on the annotation from GSK. Colours are based on the % motility reduction going from 0% (blue) and from 1 to 100% (yellow to red gradient). Some cluster examples are illustrated with proteins names above them (PLK1 is Serine/threonine-protein kinase PLK1, p38 is MAP kinase p38, VEGFR2 is Vascular endothelial growth factor receptor 2, EGFR/ErbB2 is Epidermal growth factor receptor and Receptor protein-tyrosine kinase erbB-2, GSK3 is Glycogen synthase kinase-3, IGF1R is Insulin-like growth factor I receptor). (TIF) [file pntd.0003801.s007.tif]

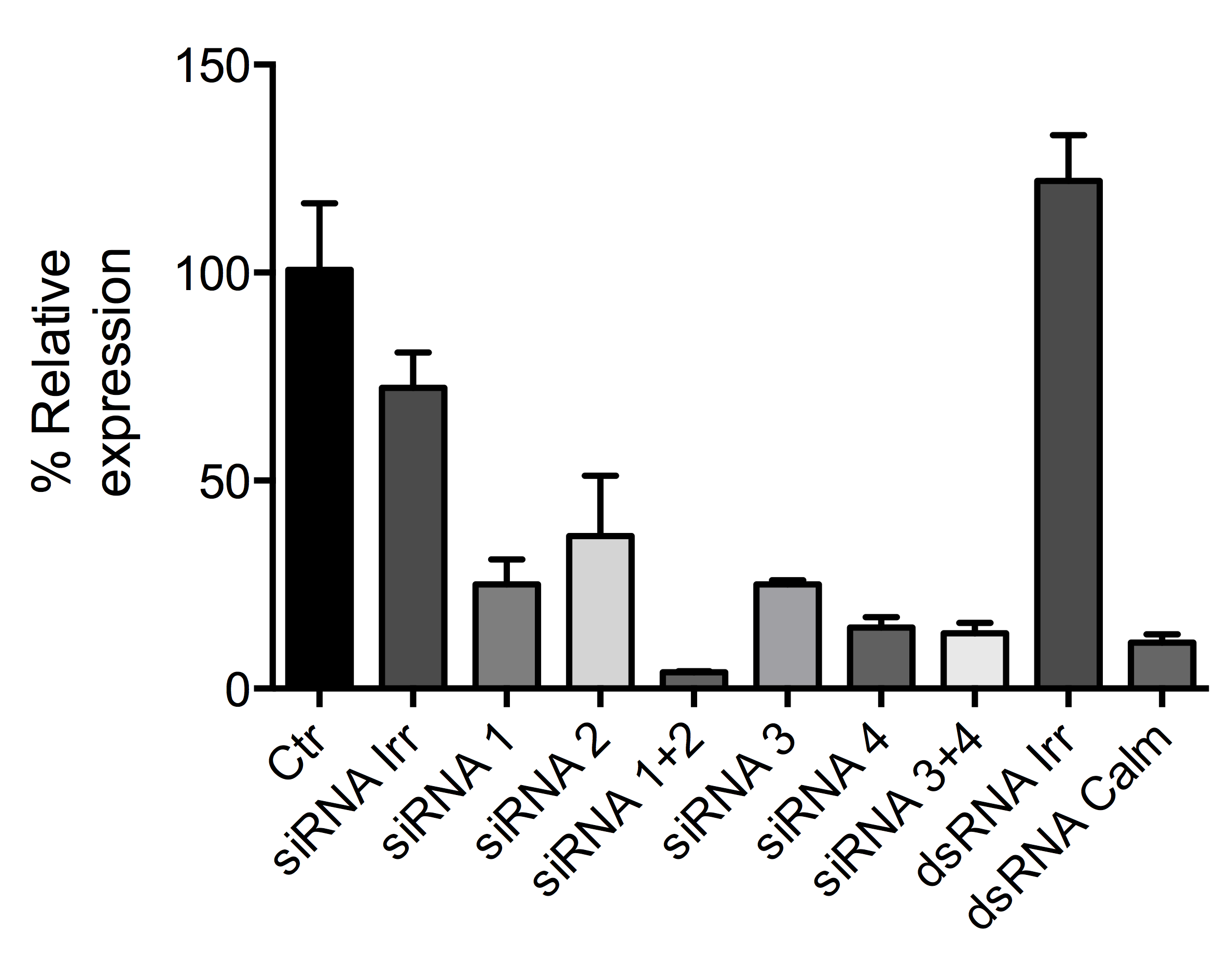

Supplement: S3 Fig — Relative expression of Sm-Calm in adult parasites electroporated with either 2.5μg/ml of synthetic siRNA (1 and 2 from Applied Biosystem, 3 and 4 from IDT) or 100μg/ml of long dsRNA and their respective IRR controls. (TIFF) [file pntd.0003801.s008.tiff]
